# Long-term care use after a stroke or femoral fracture and the role of family caregivers

**DOI:** 10.1186/s12877-020-01526-7

**Published:** 2020-04-22

**Authors:** Doutsen A. van der Burg, Maaike Diepstraten, Bram Wouterse

**Affiliations:** 1 The Dutch Healthcare Authority (NZA), Utrecht, The Netherlands; 2 The Netherlands Bureau for Economic Policy Analysis (CPB), The Hague, The Netherlands; 3 Erasmus School of Health Policy & Management, Erasmus University Rotterdam, P.O. Box 1738, 3000 DR Rotterdam, The Netherlands

**Keywords:** Long-term care, Informal care givers, Femoral fracture, Stroke

## Abstract

**Background:**

There has been a shift from institutional care towards home care, and from formal to informal care to contain long-term care (LTC) costs in many countries. However, substitution to home care or informal care might be harder to achieve for some conditions than for others. Therefore, insight is needed in differences in LTC use, and the role of potential informal care givers, across specific conditions. We analyze differences in LTC use of previously independent older patients after a fracture of femur and stroke, and in particular examine to what extent having a partner and children affects LTC use for these conditions.

**Methods:**

Using administrative data on Dutch previously independent older people (55+) with a fracture of femur or stroke in 2013, we investigate their LTC use in the year after the condition takes place. We use administrative treatment data to select individuals who were treated by a medical specialist for a stroke or femoral fracture in 2013. Subsequent LTC use is measured as using no formal care, home care, institutional care or being deceased at 13 consecutive four-weekly periods after initial treatment. We relate long-term care use to having a partner, having children, other personal characteristics and the living environment.

**Results:**

The probability to use no formal care 1 year after the initial treatment is equally high for both conditions, but patients with a fracture are more likely to use home care, while patients with a stroke are more likely to use institutional care or have died. Having a spouse has a negative effect on home care and institutional care use, but the timing of the effect, especially for institutional care, differs strongly between the two conditions. Having children also has a negative effect on formal care use, and this effect is consistently larger for patients with a fracture than patients with a stroke.

**Conclusion:**

As the condition and the effect of potential informal care givers matter for subsequent long-term care use, policy makers should take the expected prevalence of specific conditions within the older people population into account when designing long-term care policies.

## Background

In many countries, long-term care (LTC) is under pressure due to ageing populations and limited public budgets. This has led governments to stimulate older people to live independently for as long as possible. As a result, there has been a shift from institutional care towards home care [1, 2], and from formal to informal care [3]. However, substitution to home care or informal care might be harder to achieve for some conditions than for others. Therefore, insight is needed in differences in LTC use, and the role of potential informal care givers, across specific conditions.

So far, most studies focus on the determinants of LTC use for the total population of older people [4–8]. Having a spouse and having a child are positively related to using less formal care [7, 9, 10]. Also the level of disability, health status, age, gender, household size and the social network are important determinants of the use of care [4, 6–16].

Others consider the determinants of nursing home use for one single disease [17–19]. Van Rensbergen and Nawrot (2010) [20] and Rapp et al. (2015) [21] consider differences in nursing home admissions across several acute conditions. They find that some conditions, e.g. dementia and stroke [20], are stronger predictors of institutional care use than others, but they do not relate these differences to differences in the use of home care and the role of family caregivers. Wong et al. (2010) [7] investigate the effect of different conditions on the discharge probability to both home care and institutional care use among hospitalized patients. They find that discharge to home care or instituiontal care depends on the diagnosis. For example, a person with a fracture of the lower leg is more likely to be discharged to home care, while a person with a cerebrovascular disease is more likely to be discharged to institutional care. They also find that having a spouse or having children lowers the probability of both home care and institutional care use, but they do not consider how these family caregiving effects are related to specific diseases.

In this study, we focus on two severe and acute conditions, for which we can expect a strong effect on need for LTC, but for which the care trajectories, the type of care needed, and the extent to which formal care can be substituted by informal care are likely to be different. Femoral fracture and stroke are both severe acute conditions that have a lasting impact on the functioning and wellbeing of older patients, and are strong predictors of LTC use [7, 21]. At the same time, stroke seems to be associated more strongly with functional limitations than femoral fractures [22]. As a result, institutionalization rates for (female) stroke patients seem to be higher than for patients with a femoral fracture [21]. Besides, health benefits of different types of LTC are different for both conditions [23–25]. For instance, Kramer et al. [23] find, for the U.S., that admission to an inpatient rehabilitation facility instead of a skilled nursing facility has benefits for stroke patients but not for patients with a hip fracture, although Buntin et al. (2010) find benefits for both groups of patients [25]. The different impact of both conditions on disability and the types of formal care, is likely to also affect the role of informal caregivers. Bonsang (2009), for instance, found that whether informal care provided by the children is a substitute for formal care depends on the type of care needed and the level of disability [26].

We investigate whether there are differences in LTC use of previously independent older patients after a fracture of femur and a stroke. We first investigate whether both conditions are associated with different levels of home care and nursing home use at different points in time after initial treatment, controlling for an extensive set of possible confounders. Second, we examine to what extent having a partner and having children affect long-term care use for both conditions.

## Methods

### Study population

We study the use of LTC by Dutch older people who were treated by a medical specialist for stroke or a femoral fracture, during 13 consecutive four-weekly periods after initial treatment. Our source data is administrative data on all Dutch older people (55 years or older), from which we select individuals who were treated for one of these two conditions in 2013.

The Netherlands has one of the most extensive collective LTC arrangements in the world [27]. In our study period (2013–2014), a social insurance, called the exceptional medical expenses act (AWBZ), covered a broad range of both home care services (social support, personal care, nursing) and institutional care (nursing homes and residential care). Eligibility for LTC was determined by an independent assessment agency, based on health, limitations, and other relevant circumstances, such as the availability of informal care.

### Data sources

We combine data on an individual level from different administrative sources to construct our dataset. These data have been collected by Statistics Netherlands and can be linked using a personalized identification number. To identify individuals who were treated for a femoral fracture or stroke, we use a dataset containing all declarations for treatments of medical specialists financed by the basic health care insurance in 2013. The data contain *Diagnosis Treatment Combination* codes (DTCs, comparable to DRGs) that include the diagnosis. This data contains information on duration, type of care and diagnosis on declarable sub-trajectories within the whole of treatments a patient receives for a particular diagnosis. The basic insurance is compulsory and covers all Dutch inhabitants.

To measure care, we use data on LTC use in 2013 and 2014 from the Dutch Central Administrative Office (CAK). The data include information on all publicly financed formal LTC use in the Netherlands. We also include data on mortality records.

We include a number of additional datasets that contain relevant information on confounding factors. Gender, age, ethnicity, and household composition are obtained from the Dutch population register. Based on the address on January 1, 2013, we include information on the municipality of residence. We also include data on the accessibility of the home for individuals with mobility problems. We use data from the tax services to obtain gross income, net financial wealth, and net housing wealth. To control for health, we include information on eligibility for LTC 14 days before treatment. We also include total curative health care costs in 2012 (based on administrative data for the basic health insurance).

### Sample selection

To select individuals who were treated by a medical specialist for stroke or femoral fracture, we use the DTC-subtrajectory codes. These codes consists of 12 digits, providing information on the treating specialism, the diagnosis group and sub-group. The last four digits contain information on the disease. Based on this last part, we select individuals with a fracture of femur, or a hemorrhagic or ischemic stroke. We exclude patients with a transient ischemic attack (TIA), to have a more homogenous sample of patients with relatively severe strokes. As we are interested in LTC use after initial treatment, we exclude follow-up treatment, inter colleague consults, and DTCs that already started in 2012. We also exclude individuals who already use formal home and institutional care in the period just before treatment.

We start with 21,000 (44,000) DTCs for fractural femur (stroke) in 2013. This is comparable to the number of cases reported by Statistics Netherlands [28]. We lose 6.000 (9000) observations because of missing variables in other datasets and exclude 2000 (1000) patients who used institutional care at the day of the event and 5000 (8000) patients who used formal home care in the period prior to the event. As a result, we retain 7884 (26,150) observations for fractural femur (stroke).

### Outcomes

We consider four outcomes at 13 consecutive four-weekly periods after the DTC is opened: no LTC, home care, institutional care, and death. For each period, we identify whether the individual used any care or died after *T* periods (*T* = 1, 2..13). For institutional care we observe the exact date of use. Home care use is only registered on a monthly basis. As a result, we assume that someone uses home care after *T* periods if he uses home care in that month. When an individual uses both types of care at the same day, the outcome is set to institutional care. When an individual dies at a day he uses formal care, the outcome is always set to death.

Institutional care is provided in a nursing or residential care home. The setting and intensity differ depending on the needs and health problems. Nursing homes provide intensive skilled care and medical treatment to older individuals with severe health and psychogeriatric problems. Autonomy is very limited. In residential care homes, the focus is on providing assistance to older people who cannot live independently. Generally, these homes have small apartments where people live on their own or with their partners. People still have substantial autonomy. Rehabilitative care is not included, as this falls under curative care in the Dutch system.

Home care is formal care, provided by professionals, at home. This includes social support, personal care (assistance with washing, dressing and eating) and nursing. The quantity and intensity of care may vary considerably according to the needs of the elderly: from 1 hour of personal care per week to around-the-clock nursing.

### Confounders

Table 1 contains the descriptive statistics for the confounders. Health care costs, income, and wealth are measured in euros. For each variable, we construct quintiles and include dummies based on these quintiles in the regression analyses. To measure municipality size, we created 7 dummies based on the number of inhabitants. The first group is equal to 1 if the number of inhabitants > 250.000, and group 7 equals 1 if the number of inhabitants < 25.000. Urbanity is based on the address density of the area and comprises of 5 categories. The first group has value 1 if there are more than 2.500 addresses per km^2^, and the last group equals 1 if there are less than 500 addresses per km^2^.

**Table 1:** Descriptive statistics for both samples

	Fracture of femur (*n* = 7884)		Stroke (*n* = 26,150)	
	Mean	Standard deviation	Mean	Standard deviation
Partner^*^	0.541		0.669	
Having children^*^	0.842		0.872	
Man^*^	0.348		0.568	
Age	76.341	9.832	72.301	9.011
Number of children	2.187	1.589	2.209	1.474
Children living in the household^*^	0.093		0.114	
First generation immigrant^*^	0.050		0.089	
Second generation immigrant^*^	0.050		0.055	
Municipality size 1^*^	0.124		0.115	
Municipality size 2^*^	0.020		0.024	
Municipality size 3^*^	0.075		0.069	
Municipality size 4^*^	0.093		0.099	
Municipality size 5^*^	0.202		0.199	
Municipality size 6^*^	0.306		0.318	
Municipality size 7^*^	0.179		0.176	
Urbanity category 1^*^	0.214		0.195	
Urbanity category 2^*^	0.265		0.269	
Urbanity category 3^*^	0.178		0.194	
Urbanity category 4^*^	0.174		0.175	
Urbanity category 5^*^	0.170		0.167	
Accessibility home: 0/3 stars^*^	0.106		0.104	
Accessibility home: 0 stars^*^	0.028		0.025	
Accessibility home: 2 stars^*^	0.529		0.591	
Accessibility home: 3 stars^*^	0.336		0.280	
Eligibility for long-term care t-14^*^	0.090		0.048	
Healthcare costs (in euros) in 2012	5917	11,682	5509	11,375
Gross income (in euros) in 2012	46,313	42,053	50,087	45,055
Financial wealth (in euros) in 2012	224,558	422,025	177,861	391,285
Value of the house (in euros) January 2012	161,500	195,706	162,677	194,586

Descriptive statistics of the two samples

*Dichotomous variables (0 = no/1 = yes). Means are the fractions of the population

Accessibility of the home is measured in 4 categories based on the need to climb stairs. A zero star house is poorly accessible as one has to climb the stairs to reach the front door. The front door of a 2 star house can be reached without climbing the stairs. This type of house consists of multiple levels and it costs less than 10.000 euro to place a stair lift. A 3-star house is most accessible as it consists of only one floor and is accessible without climbing the stairs. For apartments in a building without an elevator it is not always clear whether the apartment is on the ground floor (3 star house) or above (0 star house). Therefore, all apartments are included in the category 0/3-mix. We include three dummies in the regressions analysis with 0-star houses being the reference category.

### Statistical analysis

For both diseases, we run 13 separate multinomial logit models; one for each period. These models estimate the log odds of using a particular type of care (or being deceased) in a particular period after treatment compared to using no care in that period. We report two types of outcomes. First, we compare the use of LTC between older people with the two different conditions, controlling for differences in confounders between the two populations. Using the estimated parameters, we make predictions for LTC use in each period for both conditions for a reference person with the same characteristics (woman aged 80, with a spouse, 2 children (not living at home), who has average healthcare costs and average wealth, and who lives in a 2-stars home (which she does not own) in an average sized municipality and city). Second, we focus on the differences in the effect of two confounders (having a spouse and having children) on the use of LTC between the two conditions. We report relative risk ratios for these confounders for both conditions. We also show the average marginal effects: the effect of a one unit increase in the confounder on the probability of the outcome, averaged over all individuals in the population.

## Results

Table 1 presents descriptive statistics for both samples separately. In both samples, most patients have a spouse, with the proportion of patients with a partner being higher in the stroke sample. Moreover, approximately 85% of the patients have children in both samples. Older people with a fracture of femur are more often female than male, while the opposite is true for the stroke sample. Besides, older people with a fracture of femur are on average older than patients with a stroke, but are similar in terms of the living environment.

### Care use after a fracture of femur and stroke

**Fig. 1:**
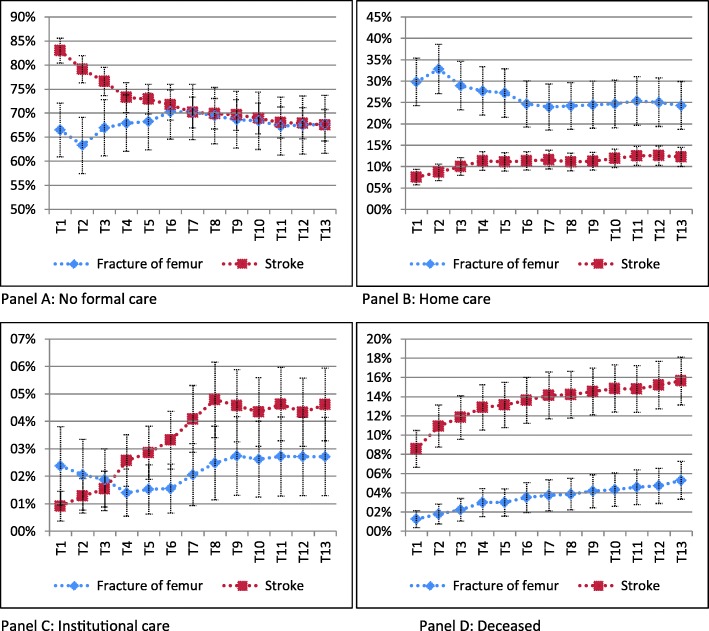
Care use of a woman aged 80, with a spouse, 2 children(not living at home), who has average healthcare costs and average wealth, and who lives in a 2‐stars living (which she does not own) in an average sized municipality and city after a fracture of femur (blue line) and a stroke (red line) up till one year after the condition takes place

Figure 1 shows the predicted outcomes for the reference person having one of the two conditions. The underlying regression tables can be found in Tables A1 and A2 in Additional File 1. At all moments in time, the likelihood to use home care after a fracture of femur is higher than after a stroke (Panel B). Shortly after the condition takes place, there is not much difference in the probability to use institutional care (Panel C). For example, three periods after a stroke her probability to receive institutional care is 1.5% while this is 1.9% after a fracture of femur. With time, the probability to use institutional care increases for the stroke patient while it stays constant for the patient with a fracture of femur. Moreover, mortality is higher in the first year after a stroke than after a femural fracture.

### The effects of having a partner and children on care use

Table 2 shows relative risk ratios for having a spouse and having children at 12, 24, and 52 weeks after a fracture of femur and stroke. For both conditions, having a partner and having children decrease the probability of using home care and institutional care. Having a spouse is also associated with lower mortality for stroke patients, and having children is associated with lower mortality for both conditions.

**Table 2:** Relative risk ratios

		Having a partner			Having children		
		*T* = 12	*T* = 24	*T* = 52	*T* = 12	*T* = 24	*T* = 52
Fracture of femur	Home care	0.622***	0.679***	0.695***	0.839*	0.732***	0.762**
	Institutional care	0.519***	0.687***	0.778*	0.840	0.605***	0.537***
	Deceased	0.841	0.893	0.878	0.678**	0.626***	0.713**
Stroke	Home care	0.596***	0.575***	0.632***	0.886	0.852**	0.790***
	Institutional care	0.612***	0.531***	0.514***	0.789*	0.743***	0.710***
	Deceased	0.891**	0.845***	0.820***	0.875*	0.844**	0.839**

*Relative risk ratios of having* a spouse and having children at 12, 24 and 52 weeks after a fracture of femur and stroke. *** *p* < 0.01, ** *p* < 0.05, * *p* < 0.1

**Fig. 2:**
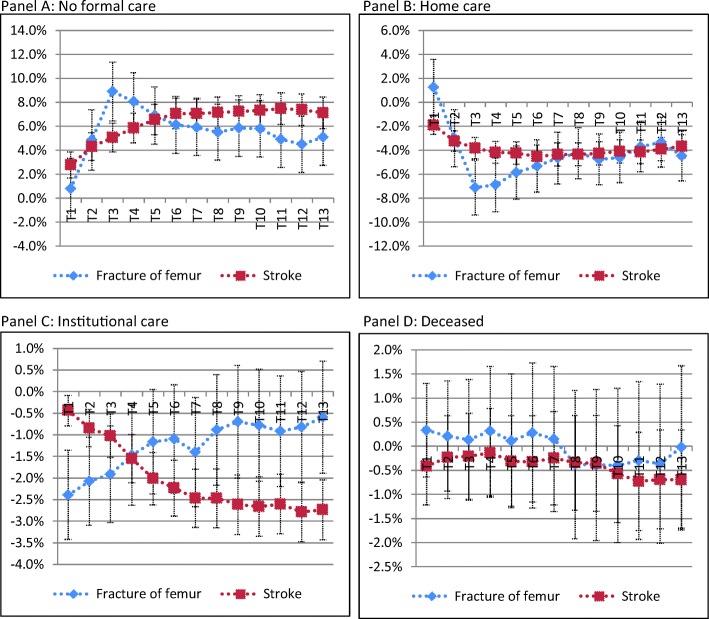
Marginal effects of having a partner after a fracture of femur (blue line) and stroke (red line) up till one year after the condition takes place

**Fig. 3:**
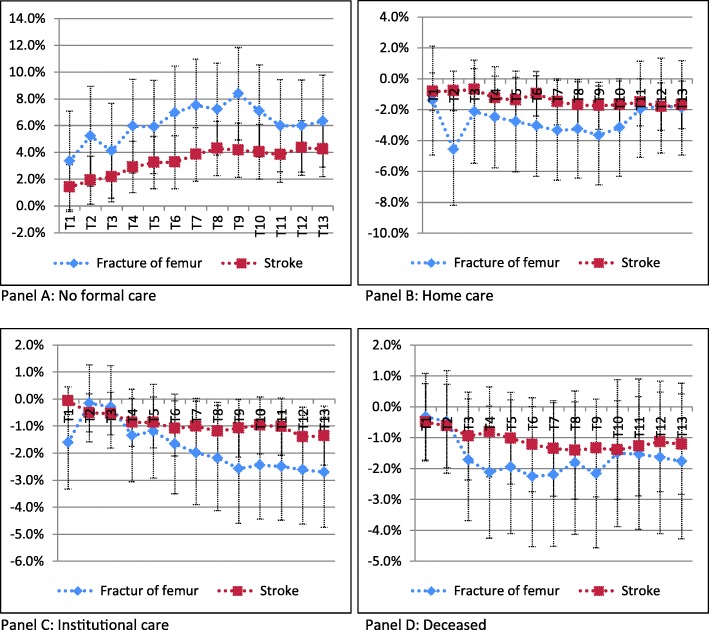
Marginal effects of having children after a fracture of femur (blue line) and stroke (red line) up till one year after the condition takes place

To gain more insight in the timing of the effects, we consider the marginal effects in Figs. 2 and 3. Initially, having a spouse has a positive effect on home care use after a fracture and a negative effect after a stroke. After 3 months, the effect is negative for both conditions, but larger for fractures. In the longer run, having a spouse has a small negative effect, equal for both conditions. Having a spouse has a negative effect on institutional care use in the first periods after a fracture, but this effect dies out over time. The time pattern for stroke is the reverse.

Having children has a consistently negative effect on both types of formal care use over time. The effect is also consistently stronger for patients with a fracture than for patients with a stroke.

## Discussion

We have assessed the impact of fracture of femur and stroke on home care and institutional care use in the first year after diagnosis for previously independent older people. We have also analyzed difference in the effects of having a spouse and having children between the conditions. The main strength of the study is that we use administrative data on all Dutch patients with a stroke and femoral fracture in 2013, and that we link this data to an extensive set of confounders.

We find that, after 1 year, the probability to use no formal care is equally high for both conditions, but patients with a fracture are more likely to use home care, while patients with a stroke are more likely to use institutional care or have died. Having a spouse has a negative effect on home care and institutional care use, but the timing of the effect, especially for institutional care, differs strongly between the two conditions. Having children also has a negative effect on formal care use, and this effect is consistently larger for patients with a fracture than patients with a stroke.

For as far as we can compare our results to other studies, our findings seem to be in line with earlier findings. Both acute conditions have been established as strong predictors of LTC use [7, 21]. Rapp et al. (2015) [21] compare institutional care use 6 months after different conditions. They also find that the likelihood of the use of institutional care is higher after a stroke than after a fracture of femur, especially for women. This difference might indicate that, on average, limitations after a stroke are more severe than after a fracture [22].

The negative effect of having a spouse and children on LTC use is also well established for the general older population [9, 10] and after hospitalization [7]. What is novel in our study, is that we condition these effects on having a particular condition, and that we find that they indeed differ. What exactly explains the difference in the effect of family caregivers between the two conditions requires additional research. A higher severity of limitations after a stroke might explain why there is more scope for a partner to prevent a nursing home admission for those patients than for patient with a fracture. However, this would not explain why the effect of children is stronger for patients with a fracture than with a stroke. There is heterogeneity in the type of care that is required across different types of conditions [29]. It might be that children are less willing or able to provide the type of informal care required for stroke patients than that for fracture patients.

The study also has limitations. First, we have only included two conditions. We do this because these two conditions are acute and can be expected to have a direct impact on care use. Dementia is another important condition, where the timing of care use and the impact of family caregivers could be quite different, but the onset of that condition is much more gradual and much harder to identify in administrative data. Second, our study is in the context of the Dutch care system of 2013. The Dutch LTC system is very extensive compared to most countries [27], and eligibility for care, partly, depends on policy rules specific to the Netherlands. This means that caution is needed in generalizing the finding to other countries, or even to the current Dutch system (that was reformed in 2015). A specific issue is that geriatric rehabilitation facility care, which one might consider LTC, is actually part of curative care in the Netherlands, and thus not included. Third, the fact that we sometimes find significant effects of having a spouse or children on mortality, might indicate that these variables are correlated with unobserved health, or it might reflect the quality of informal caregiving at home. Caution is thus again needed in interpreting the effects as causal.

## Conclusion

This study shows that the LTC trajectory and the role of family caregivers after a hospital admission of an older individual can differ considerably across diseases. These differences are important for policy makers who try to stimulate aging in place. Policies to stimulate the provision of informal care instead of formal care, or home care instead of nursing home care might be more effective for patients with some diseases than for others. The extent to which ageing in place will be able to contribute to containing the costs of LTC will thus depend strongly on the (future) disease burden of the older population.

## Supplementary information

**Additional file 1: **Regression coefficients. Contains the **Tables A1.** and **A2.** of the regression estimates underlying the reported results in the paper.

## Data Availability

The data that support the findings of this study are available from Statistics Netherlands (CBS) but restrictions apply to the availability of these data, which were used under license for the current study, and so are not publicly available. Data are however available from the authors upon reasonable request and with permission of Statistics Netherlands (CBS).

## Abbreviations

AWBZ: Exceptional medical expenses act
CAK: Central Administrative Office
CBS: Statistics Netherlands
DTC: Diagnosis Treatment Combination
LTC: Long Term Care
TIA: transient ischemic attack
