# Supplementary material for: Long-term care use after a stroke or femoral fracture and the role of family caregivers
Source: BMC Geriatr. 2020 Apr 22;20:150. doi: 10.1186/s12877-020-01526-7 (PMC7178980; doi:10.1186/s12877-020-01526-7)
Supplement: Supplementary file 1 — Additional file 1: Regression coefficients. Contains the Tables A1. and A2. of the regression estimates underlying the reported results in the paper. [file 12877_2020_1526_MOESM1_ESM.docx]

**Regression coefficients**

A.1 Fracture of femur (N=7,884)

|  | T=4 | T=4 | T=4 | T=8 | T=8 | T=8 | T=12 | T=12 | T=12 |
| --- | --- | --- | --- | --- | --- | --- | --- | --- | --- |
|  | Home care | Institutional care | Being deceased | Home care | Institutional care | Being deceased | Home care | Institutional care | Being deceased |
| Healthcare costs quintile 2 | 0.230*** | 0.186 | 0.114 | 0.272*** | 0.018 | -0.024 | 0.176* | 0.265 | -0.197 |
|  | (0.09) | (0.20) | (0.26) | (0.09) | (0.23) | (0.20) | (0.09) | (0.22) | (0.19) |
| Healthcare costs quintile 3 | 0.168* | 0.106 | 0.237 | 0.389*** | 0.178 | -0.043 | 0.368*** | 0.251 | -0.151 |
|  | (0.09) | (0.20) | (0.25) | (0.09) | (0.22) | (0.20) | (0.09) | (0.22) | (0.18) |
| Healthcare costs quintile 4 | 0.095 | 0.242 | 0.578** | 0.335*** | 0.453** | 0.379** | 0.386*** | 0.448** | 0.301* |
|  | (0.09) | (0.20) | (0.23) | (0.09) | (0.21) | (0.19) | (0.09) | (0.22) | (0.17) |
| Healthcare costs quintile 5 | 0.321*** | 0.394** | 0.517** | 0.488*** | 0.895*** | 0.490*** | 0.629*** | 1.344*** | 0.546*** |
|  | (0.09) | (0.20) | (0.23) | (0.09) | (0.20) | (0.19) | (0.09) | (0.20) | (0.17) |
| Eligibility for long-term care t-14 | -0.461*** | 0.750*** | 0.817*** | -0.278*** | 0.680*** | 1.007*** | -0.042 | 0.866*** | 1.055*** |
|  | (0.11) | (0.15) | (0.16) | (0.10) | (0.16) | (0.14) | (0.10) | (0.15) | (0.14) |
| Man | -0.376*** | -0.166 | 0.679*** | -0.348*** | -0.018 | 0.778*** | -0.231*** | 0.066 | 0.790*** |
|  | (0.06) | (0.14) | (0.15) | (0.06) | (0.14) | (0.12) | (0.06) | (0.13) | (0.11) |
| Partner | 0.035 | -0.654*** | 0.081 | -0.201*** | -0.677*** | -0.056 | -0.475*** | -0.655*** | -0.174 |
|  | (0.06) | (0.15) | (0.16) | (0.06) | (0.15) | (0.14) | (0.07) | (0.14) | (0.13) |
| Children living in the household | -0.109 | -0.214 | 0.075 | -0.045 | -0.164 | 0.041 | -0.177 | -0.123 | -0.020 |
|  | (0.11) | (0.27) | (0.25) | (0.11) | (0.27) | (0.22) | (0.12) | (0.25) | (0.20) |
| Number of children | 0.042** | 0.054 | -0.014 | 0.061*** | 0.006 | -0.017 | 0.036* | -0.067 | -0.003 |
|  | (0.02) | (0.04) | (0.05) | (0.02) | (0.04) | (0.04) | (0.02) | (0.04) | (0.04) |
| Having children | -0.109 | -0.424** | -0.164 | -0.259*** | -0.155 | -0.208 | -0.175* | -0.175 | -0.388** |
|  | (0.09) | (0.19) | (0.23) | (0.09) | (0.20) | (0.19) | (0.10) | (0.18) | (0.17) |
| First generation immigrant | 0.014 | -0.017 | -0.425 | 0.132 | 0.008 | -0.291 | 0.219* | 0.092 | -0.369 |
|  | (0.13) | (0.25) | (0.32) | (0.12) | (0.26) | (0.27) | (0.13) | (0.23) | (0.25) |
| Second generation immigrant | -0.187 | -0.629* | 0.184 | -0.125 | -0.341 | 0.051 | 0.018 | -0.363 | 0.019 |
|  | (0.13) | (0.35) | (0.29) | (0.12) | (0.32) | (0.25) | (0.13) | (0.31) | (0.24) |
| Age | 0.015*** | 0.046*** | 0.101*** | 0.036*** | 0.070*** | 0.094*** | 0.056*** | 0.101*** | 0.098*** |
|  | (0.00) | (0.01) | (0.01) | (0.00) | (0.01) | (0.01) | (0.00) | (0.01) | (0.01) |
| Urbanity category 2 | 0.189** | -0.068 | 0.080 | 0.204** | -0.024 | -0.167 | 0.008 | -0.120 | -0.138 |
|  | (0.09) | (0.19) | (0.21) | (0.09) | (0.18) | (0.18) | (0.09) | (0.17) | (0.17) |
| Urbanity category 3 | 0.331*** | -0.065 | 0.076 | 0.246** | -0.106 | -0.016 | 0.011 | -0.330 | -0.096 |
|  | (0.10) | (0.22) | (0.25) | (0.10) | (0.22) | (0.21) | (0.11) | (0.21) | (0.19) |
| Urbanity category 4 | 0.532*** | 0.207 | 0.194 | 0.381*** | -0.094 | 0.000 | 0.236** | -0.171 | 0.043 |
|  | (0.11) | (0.24) | (0.27) | (0.11) | (0.25) | (0.23) | (0.11) | (0.23) | (0.21) |
| Urbanity category 5 | 0.483*** | 0.157 | 0.176 | 0.355*** | -0.228 | 0.002 | 0.132 | -0.117 | 0.027 |
|  | (0.11) | (0.25) | (0.28) | (0.11) | (0.27) | (0.23) | (0.12) | (0.24) | (0.22) |
| Municipality size 2 | 0.661*** | -0.742 | 0.429 | 0.865*** | -0.269 | 0.610 | 0.387* | -0.184 | 0.491 |
|  | (0.20) | (0.61) | (0.48) | (0.20) | (0.54) | (0.42) | (0.22) | (0.46) | (0.36) |
| Municipality size 3 | 0.312** | -0.427 | -0.199 | 0.394*** | -0.403 | 0.099 | 0.357** | -0.351 | -0.176 |
|  | (0.14) | (0.29) | (0.34) | (0.13) | (0.29) | (0.29) | (0.14) | (0.26) | (0.26) |
| Municipality size 4 | 0.441*** | 0.040 | 0.069 | 0.624*** | 0.418* | 0.098 | 0.545*** | 0.369* | -0.160 |
|  | (0.13) | (0.25) | (0.30) | (0.13) | (0.23) | (0.28) | (0.13) | (0.22) | (0.25) |
| Municipality size 5 | 0.313*** | -0.172 | -0.083 | 0.405*** | -0.218 | 0.329 | 0.432*** | -0.304 | 0.148 |
|  | (0.12) | (0.22) | (0.26) | (0.11) | (0.22) | (0.22) | (0.12) | (0.20) | (0.20) |
| Municipality size 6 | 0.302** | -0.091 | 0.200 | 0.460*** | -0.116 | 0.445* | 0.507*** | -0.255 | 0.250 |
|  | (0.12) | (0.23) | (0.27) | (0.12) | (0.23) | (0.23) | (0.12) | (0.22) | (0.21) |
| Municipality size 7 | 0.212 | -0.151 | 0.127 | 0.411*** | 0.060 | 0.556** | 0.502*** | -0.059 | 0.339 |
|  | (0.13) | (0.26) | (0.30) | (0.13) | (0.27) | (0.26) | (0.14) | (0.25) | (0.24) |
| Gross income quintile 2 | 0.142 | -0.083 | -0.051 | -0.069 | -0.157 | -0.114 | -0.111 | -0.223 | -0.113 |
|  | (0.09) | (0.18) | (0.22) | (0.09) | (0.19) | (0.18) | (0.09) | (0.17) | (0.17) |
| Gross income quintile 3 | -0.133 | 0.033 | -0.276 | -0.186** | 0.051 | -0.188 | -0.108 | -0.157 | -0.121 |
|  | (0.09) | (0.18) | (0.23) | (0.09) | (0.19) | (0.19) | (0.10) | (0.18) | (0.18) |
| Gross income quintile 4 | -0.224** | -0.443** | -0.242 | -0.399*** | -0.390* | -0.317 | -0.393*** | -0.374* | -0.242 |
|  | (0.10) | (0.23) | (0.25) | (0.10) | (0.23) | (0.21) | (0.11) | (0.21) | (0.19) |
| Gross income quintile 5 | -0.371*** | -0.448* | 0.122 | -0.595*** | -0.344 | 0.017 | -0.460*** | -0.305 | 0.043 |
|  | (0.12) | (0.26) | (0.28) | (0.12) | (0.27) | (0.24) | (0.13) | (0.25) | (0.22) |
| Home ownership | 0.003 | 0.055 | 0.896*** | 0.035 | -0.043 | 0.967*** | 0.092 | 0.033 | 0.822*** |
|  | (0.09) | (0.22) | (0.24) | (0.10) | (0.24) | (0.21) | (0.10) | (0.23) | (0.19) |
| Value of the house | 0.000 | -0.000 | -0.000*** | -0.000 | -0.000 | -0.000*** | -0.000*** | -0.000** | -0.000*** |
|  | (0.00) | (0.00) | (0.00) | (0.00) | (0.00) | (0.00) | (0.00) | (0.00) | (0.00) |
| Financial wealth quintile 2 | -0.124 | -0.080 | -0.051 | -0.022 | 0.074 | -0.093 | 0.054 | 0.187 | -0.153 |
|  | (0.08) | (0.18) | (0.23) | (0.08) | (0.19) | (0.18) | (0.09) | (0.19) | (0.17) |
| Financial wealth quintile 3 | -0.203** | -0.107 | -0.111 | -0.120 | -0.149 | -0.287 | 0.062 | 0.155 | -0.244 |
|  | (0.09) | (0.19) | (0.23) | (0.09) | (0.20) | (0.19) | (0.09) | (0.19) | (0.17) |
| Financial wealth quintile 4 | -0.247*** | -0.148 | 0.618*** | -0.145 | 0.069 | 0.514*** | -0.063 | 0.326 | 0.446*** |
|  | (0.09) | (0.21) | (0.22) | (0.09) | (0.21) | (0.18) | (0.10) | (0.20) | (0.16) |
| Financial wealth quintile 5 | 0.005 | -0.080 | -2.956*** | 0.120 | 0.015 | -2.718*** | 0.065 | 0.320 | -2.895*** |
|  | (0.10) | (0.23) | (0.55) | (0.10) | (0.25) | (0.40) | (0.11) | (0.24) | (0.37) |
| Accessibility home: 0/3 stars | 0.057 | -0.282 | -0.098 | 0.071 | 0.030 | 0.253 | 0.159 | 0.635* | 0.287 |
|  | (0.19) | (0.33) | (0.39) | (0.18) | (0.34) | (0.37) | (0.19) | (0.35) | (0.34) |
| Accessibility home: 2 stars | -0.015 | -0.270 | -0.152 | -0.071 | -0.098 | 0.062 | 0.055 | 0.487 | 0.199 |
|  | (0.18) | (0.31) | (0.37) | (0.17) | (0.32) | (0.35) | (0.18) | (0.34) | (0.33) |
| Accessibility home: 3 stars | 0.105 | -0.183 | -0.398 | 0.122 | -0.057 | 0.051 | 0.218 | 0.517 | 0.231 |
|  | (0.18) | (0.30) | (0.37) | (0.17) | (0.31) | (0.35) | (0.17) | (0.33) | (0.32) |
| Constant | -2.508*** | -5.561*** | -11.660*** | -3.849*** | -7.890*** | -10.606*** | -5.548*** | -10.755*** | -10.439*** |
|  | (0.32) | (0.69) | (0.86) | (0.32) | (0.74) | (0.72) | (0.34) | (0.75) | (0.66) |
|  |  |  |  |  |  |  |  |  |  |
| Pseudo R squared | 0.0664 | 0.0664 | 0.0664 | 0.0957 | 0.0957 | 0.0957 | 0.128 | 0.128 | 0.128 |
| Log likelihood | -6368 | -6368 | -6368 | -6574 | -6574 | -6574 | -6437 | -6437 | -6437 |

Regression coefficients for the sample fracture of femur at *T* weeks after the condition took place. *** p<0.01, ** p<0.05, * p<0.1.

|  | T=16 | T=16 | T=16 | T=20 | T=20 | T=20 | T=24 | T=24 | T=24 |
| --- | --- | --- | --- | --- | --- | --- | --- | --- | --- |
|  | Home care | Institutional care | Being deceased | Home care | Institutional care | Being deceased | Home care | Institutional care | Being deceased |
| Healthcare costs quintile 2 | 0.175* | 0.217 | -0.246 | 0.236** | 0.057 | -0.208 | 0.185* | 0.048 | -0.239 |
|  | (0.10) | (0.22) | (0.18) | (0.10) | (0.21) | (0.17) | (0.10) | (0.20) | (0.17) |
| Healthcare costs quintile 3 | 0.378*** | 0.279 | -0.125 | 0.459*** | 0.289 | -0.139 | 0.364*** | 0.211 | -0.134 |
|  | (0.10) | (0.22) | (0.17) | (0.10) | (0.20) | (0.17) | (0.10) | (0.19) | (0.16) |
| Healthcare costs quintile 4 | 0.410*** | 0.552*** | 0.338** | 0.521*** | 0.391* | 0.365** | 0.475*** | 0.293 | 0.368** |
|  | (0.10) | (0.21) | (0.16) | (0.10) | (0.20) | (0.16) | (0.10) | (0.19) | (0.15) |
| Healthcare costs quintile 5 | 0.680*** | 1.440*** | 0.646*** | 0.782*** | 1.345*** | 0.709*** | 0.815*** | 1.319*** | 0.802*** |
|  | (0.10) | (0.20) | (0.16) | (0.10) | (0.18) | (0.15) | (0.10) | (0.18) | (0.15) |
| Eligibility for long-term care t-14 | -0.006 | 0.883*** | 1.113*** | 0.036 | 1.079*** | 1.252*** | 0.091 | 1.054*** | 1.242*** |
|  | (0.10) | (0.15) | (0.13) | (0.11) | (0.14) | (0.12) | (0.11) | (0.14) | (0.12) |
| Man | -0.202*** | 0.006 | 0.754*** | -0.128* | 0.020 | 0.797*** | -0.104 | 0.074 | 0.768*** |
|  | (0.07) | (0.13) | (0.11) | (0.07) | (0.13) | (0.10) | (0.07) | (0.12) | (0.10) |
| Partner | -0.472*** | -0.529*** | -0.137 | -0.418*** | -0.418*** | -0.148 | -0.388*** | -0.375*** | -0.114 |
|  | (0.07) | (0.14) | (0.12) | (0.07) | (0.14) | (0.11) | (0.07) | (0.13) | (0.11) |
| Children living in the household | -0.128 | -0.100 | -0.041 | -0.029 | 0.187 | 0.005 | -0.132 | 0.015 | 0.064 |
|  | (0.12) | (0.25) | (0.19) | (0.13) | (0.23) | (0.19) | (0.13) | (0.23) | (0.18) |
| Number of children | 0.033 | -0.022 | 0.015 | 0.027 | -0.030 | -0.009 | 0.043* | -0.021 | -0.012 |
|  | (0.02) | (0.04) | (0.04) | (0.02) | (0.04) | (0.04) | (0.02) | (0.04) | (0.03) |
| Having children | -0.246** | -0.443** | -0.463*** | -0.264*** | -0.400** | -0.418*** | -0.312*** | -0.502*** | -0.469*** |
|  | (0.10) | (0.18) | (0.16) | (0.10) | (0.17) | (0.16) | (0.10) | (0.17) | (0.15) |
| First generation immigrant | 0.187 | 0.011 | -0.284 | 0.085 | -0.123 | -0.474** | 0.061 | -0.137 | -0.465** |
|  | (0.13) | (0.23) | (0.23) | (0.13) | (0.23) | (0.23) | (0.14) | (0.22) | (0.22) |
| Second generation immigrant | 0.122 | -0.385 | 0.101 | 0.083 | -0.108 | 0.014 | 0.095 | -0.249 | 0.090 |
|  | (0.13) | (0.31) | (0.22) | (0.13) | (0.27) | (0.22) | (0.13) | (0.28) | (0.20) |
| Age | 0.067*** | 0.112*** | 0.099*** | 0.073*** | 0.111*** | 0.099*** | 0.075*** | 0.121*** | 0.102*** |
|  | (0.00) | (0.01) | (0.01) | (0.00) | (0.01) | (0.01) | (0.00) | (0.01) | (0.01) |
| Urbanity category 2 | -0.009 | -0.108 | -0.155 | -0.021 | -0.181 | -0.156 | 0.002 | -0.134 | -0.111 |
|  | (0.10) | (0.17) | (0.16) | (0.10) | (0.16) | (0.15) | (0.10) | (0.16) | (0.15) |
| Urbanity category 3 | 0.032 | -0.511** | -0.121 | 0.050 | -0.572*** | -0.147 | 0.000 | -0.565*** | -0.110 |
|  | (0.11) | (0.21) | (0.18) | (0.11) | (0.21) | (0.17) | (0.11) | (0.20) | (0.17) |
| Urbanity category 4 | 0.163 | -0.384 | -0.119 | 0.180 | -0.558** | -0.117 | 0.166 | -0.564** | -0.076 |
|  | (0.12) | (0.23) | (0.20) | (0.12) | (0.23) | (0.19) | (0.12) | (0.22) | (0.18) |
| Urbanity category 5 | 0.176 | -0.335 | -0.093 | 0.237* | -0.492** | -0.097 | 0.203 | -0.344 | -0.060 |
|  | (0.12) | (0.24) | (0.21) | (0.12) | (0.24) | (0.20) | (0.13) | (0.23) | (0.19) |
| Municipality size 2 | 0.100 | -1.240** | 0.199 | -0.197 | -0.787 | 0.108 | -0.383 | -0.776* | -0.072 |
|  | (0.24) | (0.61) | (0.34) | (0.25) | (0.49) | (0.33) | (0.27) | (0.46) | (0.33) |
| Municipality size 3 | 0.331** | -0.283 | -0.148 | 0.255* | -0.175 | -0.158 | 0.332** | -0.159 | -0.030 |
|  | (0.14) | (0.24) | (0.24) | (0.15) | (0.23) | (0.23) | (0.15) | (0.23) | (0.22) |
| Municipality size 4 | 0.493*** | 0.136 | -0.218 | 0.383*** | 0.117 | -0.254 | 0.460*** | 0.061 | -0.230 |
|  | (0.14) | (0.22) | (0.24) | (0.14) | (0.22) | (0.23) | (0.14) | (0.21) | (0.22) |
| Municipality size 5 | 0.486*** | -0.412** | 0.128 | 0.301** | -0.337* | 0.071 | 0.330*** | -0.378** | 0.064 |
|  | (0.12) | (0.20) | (0.19) | (0.12) | (0.19) | (0.18) | (0.12) | (0.19) | (0.18) |
| Municipality size 6 | 0.489*** | -0.255 | 0.228 | 0.347*** | -0.114 | 0.193 | 0.385*** | -0.166 | 0.153 |
|  | (0.13) | (0.21) | (0.20) | (0.13) | (0.21) | (0.19) | (0.13) | (0.20) | (0.18) |
| Municipality size 7 | 0.510*** | 0.021 | 0.286 | 0.264* | 0.120 | 0.190 | 0.396*** | 0.071 | 0.157 |
|  | (0.14) | (0.25) | (0.22) | (0.14) | (0.24) | (0.22) | (0.14) | (0.23) | (0.21) |
| Gross income quintile 2 | -0.095 | -0.353** | -0.171 | -0.104 | -0.294* | -0.211 | -0.123 | -0.304* | -0.206 |
|  | (0.09) | (0.17) | (0.16) | (0.09) | (0.16) | (0.15) | (0.09) | (0.16) | (0.15) |
| Gross income quintile 3 | -0.200** | -0.477*** | -0.168 | -0.227** | -0.634*** | -0.222 | -0.277*** | -0.619*** | -0.280* |
|  | (0.10) | (0.19) | (0.17) | (0.10) | (0.18) | (0.16) | (0.10) | (0.18) | (0.16) |
| Gross income quintile 4 | -0.470*** | -0.624*** | -0.222 | -0.505*** | -0.665*** | -0.270 | -0.559*** | -0.650*** | -0.318* |
|  | (0.11) | (0.21) | (0.18) | (0.11) | (0.20) | (0.17) | (0.11) | (0.20) | (0.17) |
| Gross income quintile 5 | -0.470*** | -0.430* | -0.064 | -0.596*** | -0.545** | -0.055 | -0.606*** | -0.519** | -0.115 |
|  | (0.13) | (0.25) | (0.21) | (0.13) | (0.24) | (0.20) | (0.13) | (0.24) | (0.19) |
| Home ownership | 0.088 | -0.112 | 0.623*** | 0.098 | -0.158 | 0.607*** | -0.034 | -0.219 | 0.506*** |
|  | (0.11) | (0.23) | (0.18) | (0.11) | (0.23) | (0.17) | (0.11) | (0.22) | (0.16) |
| Value of the house | -0.000*** | -0.000* | -0.000*** | -0.000*** | -0.000** | -0.000*** | -0.000** | -0.000** | -0.000*** |
|  | (0.00) | (0.00) | (0.00) | (0.00) | (0.00) | (0.00) | (0.00) | (0.00) | (0.00) |
| Financial wealth quintile 2 | 0.063 | 0.219 | -0.154 | 0.080 | 0.274 | -0.141 | 0.073 | 0.264 | -0.178 |
|  | (0.09) | (0.18) | (0.15) | (0.09) | (0.17) | (0.15) | (0.10) | (0.17) | (0.14) |
| Financial wealth quintile 3 | 0.048 | 0.073 | -0.272* | 0.026 | 0.067 | -0.288* | -0.015 | -0.095 | -0.308** |
|  | (0.10) | (0.19) | (0.16) | (0.10) | (0.19) | (0.16) | (0.10) | (0.18) | (0.15) |
| Financial wealth quintile 4 | -0.054 | 0.407** | 0.469*** | -0.039 | 0.421** | 0.470*** | -0.102 | 0.360* | 0.410*** |
|  | (0.10) | (0.20) | (0.15) | (0.11) | (0.19) | (0.15) | (0.11) | (0.19) | (0.14) |
| Financial wealth quintile 5 | 0.040 | 0.413* | -2.544*** | 0.091 | 0.524** | -2.457*** | 0.106 | 0.431* | -2.356*** |
|  | (0.11) | (0.24) | (0.31) | (0.11) | (0.23) | (0.28) | (0.11) | (0.23) | (0.26) |
| Accessibility home: 0/3 stars | 0.307 | 0.473 | 0.359 | 0.359* | 0.427 | 0.334 | 0.302 | 0.353 | 0.436 |
|  | (0.19) | (0.33) | (0.32) | (0.20) | (0.30) | (0.32) | (0.20) | (0.29) | (0.31) |
| Accessibility home: 2 stars | 0.047 | 0.410 | 0.297 | 0.256 | 0.246 | 0.376 | 0.222 | 0.253 | 0.475 |
|  | (0.18) | (0.32) | (0.31) | (0.19) | (0.29) | (0.30) | (0.19) | (0.28) | (0.30) |
| Accessibility home: 3 stars | 0.183 | 0.440 | 0.219 | 0.313* | 0.272 | 0.270 | 0.258 | 0.226 | 0.389 |
|  | (0.18) | (0.31) | (0.30) | (0.19) | (0.28) | (0.30) | (0.19) | (0.27) | (0.30) |
| Constant | -6.436*** | -11.150*** | -10.187*** | -6.997*** | -10.866*** | -10.131*** | -7.075*** | -11.363*** | -10.229*** |
|  | (0.36) | (0.75) | (0.62) | (0.37) | (0.71) | (0.59) | (0.37) | (0.70) | (0.58) |
|  |  |  |  |  |  |  |  |  |  |
| Pseudo R squared | 0.144 | 0.144 | 0.144 | 0.150 | 0.150 | 0.150 | 0.156 | 0.156 | 0.156 |
| Log likelihood | -6453 | -6453 | -6453 | -6501 | -6501 | -6501 | -6587 | -6587 | -6587 |

Regression coefficients for the sample fracture of femur at *T* weeks after the condition took place. *** p<0.01, ** p<0.05, * p<0.1.

|  | T=28 | T=28 | T=28 | T=32 | T=32 | T=32 | T=36 | T=36 | T=36 |
| --- | --- | --- | --- | --- | --- | --- | --- | --- | --- |
|  | Home care | Institutional care | Being deceased | Home care | Institutional care | Being deceased | Home care | Institutional care | Being deceased |
| Healthcare costs quintile 2 | 0.197* | -0.002 | -0.214 | 0.174* | 0.066 | -0.236 | 0.197* | 0.108 | -0.214 |
|  | (0.10) | (0.19) | (0.16) | (0.11) | (0.19) | (0.16) | (0.11) | (0.19) | (0.15) |
| Healthcare costs quintile 3 | 0.365*** | 0.265 | -0.100 | 0.328*** | 0.261 | -0.152 | 0.349*** | 0.278 | -0.123 |
|  | (0.10) | (0.19) | (0.16) | (0.10) | (0.19) | (0.15) | (0.10) | (0.18) | (0.15) |
| Healthcare costs quintile 4 | 0.515*** | 0.270 | 0.432*** | 0.529*** | 0.251 | 0.409*** | 0.522*** | 0.331* | 0.453*** |
|  | (0.10) | (0.19) | (0.15) | (0.10) | (0.19) | (0.14) | (0.10) | (0.19) | (0.14) |
| Healthcare costs quintile 5 | 0.782*** | 1.212*** | 0.845*** | 0.823*** | 1.249*** | 0.862*** | 0.879*** | 1.273*** | 0.980*** |
|  | (0.10) | (0.17) | (0.14) | (0.10) | (0.17) | (0.14) | (0.11) | (0.17) | (0.14) |
| Eligibility for long-term care t-14 | 0.172 | 1.065*** | 1.334*** | 0.141 | 1.097*** | 1.361*** | 0.187 | 1.095*** | 1.329*** |
|  | (0.11) | (0.14) | (0.12) | (0.11) | (0.14) | (0.12) | (0.11) | (0.14) | (0.12) |
| Man | -0.120* | 0.133 | 0.742*** | -0.125* | 0.068 | 0.777*** | -0.096 | 0.101 | 0.770*** |
|  | (0.07) | (0.12) | (0.09) | (0.07) | (0.12) | (0.09) | (0.07) | (0.12) | (0.09) |
| Partner | -0.367*** | -0.423*** | -0.133 | -0.346*** | -0.318** | -0.182* | -0.382*** | -0.286** | -0.188* |
|  | (0.07) | (0.13) | (0.11) | (0.07) | (0.13) | (0.10) | (0.07) | (0.13) | (0.10) |
| Children living in the household | 0.061 | 0.131 | 0.080 | 0.058 | 0.012 | 0.160 | 0.015 | 0.168 | 0.148 |
|  | (0.13) | (0.23) | (0.17) | (0.13) | (0.24) | (0.16) | (0.13) | (0.23) | (0.16) |
| Number of children | 0.056** | -0.008 | -0.011 | 0.046** | -0.008 | -0.033 | 0.054** | 0.001 | -0.019 |
|  | (0.02) | (0.04) | (0.03) | (0.02) | (0.04) | (0.03) | (0.02) | (0.04) | (0.03) |
| Having children | -0.349*** | -0.562*** | -0.458*** | -0.346*** | -0.579*** | -0.398*** | -0.398*** | -0.657*** | -0.457*** |
|  | (0.11) | (0.17) | (0.15) | (0.11) | (0.17) | (0.15) | (0.11) | (0.16) | (0.14) |
| First generation immigrant | 0.108 | -0.133 | -0.329 | 0.100 | -0.122 | -0.297 | 0.111 | -0.277 | -0.256 |
|  | (0.14) | (0.22) | (0.20) | (0.14) | (0.22) | (0.20) | (0.14) | (0.23) | (0.19) |
| Second generation immigrant | 0.133 | -0.068 | 0.057 | 0.140 | -0.087 | 0.008 | 0.151 | -0.225 | -0.025 |
|  | (0.14) | (0.26) | (0.20) | (0.14) | (0.26) | (0.20) | (0.14) | (0.26) | (0.19) |
| Age | 0.078*** | 0.122*** | 0.099*** | 0.080*** | 0.120*** | 0.096*** | 0.079*** | 0.116*** | 0.095*** |
|  | (0.00) | (0.01) | (0.01) | (0.00) | (0.01) | (0.01) | (0.00) | (0.01) | (0.01) |
| Urbanity category 2 | -0.074 | -0.132 | -0.104 | -0.056 | -0.134 | -0.112 | -0.142 | -0.192 | -0.205 |
|  | (0.10) | (0.15) | (0.14) | (0.10) | (0.15) | (0.14) | (0.10) | (0.15) | (0.13) |
| Urbanity category 3 | -0.008 | -0.573*** | -0.146 | 0.041 | -0.567*** | -0.170 | -0.044 | -0.517*** | -0.225 |
|  | (0.11) | (0.20) | (0.16) | (0.12) | (0.20) | (0.16) | (0.12) | (0.19) | (0.15) |
| Urbanity category 4 | 0.105 | -0.601*** | -0.094 | 0.064 | -0.717*** | -0.158 | 0.013 | -0.634*** | -0.212 |
|  | (0.12) | (0.22) | (0.18) | (0.13) | (0.22) | (0.17) | (0.13) | (0.22) | (0.17) |
| Urbanity category 5 | 0.122 | -0.329 | -0.099 | 0.131 | -0.406* | -0.141 | 0.073 | -0.416* | -0.213 |
|  | (0.13) | (0.23) | (0.19) | (0.13) | (0.23) | (0.18) | (0.13) | (0.22) | (0.18) |
| Municipality size 2 | -0.376 | -0.652 | -0.162 | -0.314 | -0.261 | -0.188 | -0.477 | -0.258 | -0.139 |
|  | (0.27) | (0.43) | (0.32) | (0.28) | (0.38) | (0.32) | (0.30) | (0.36) | (0.30) |
| Municipality size 3 | 0.271* | -0.131 | -0.144 | 0.323** | -0.179 | -0.129 | 0.333** | -0.234 | -0.127 |
|  | (0.15) | (0.22) | (0.21) | (0.15) | (0.22) | (0.20) | (0.16) | (0.22) | (0.20) |
| Municipality size 4 | 0.461*** | 0.035 | -0.342 | 0.472*** | -0.004 | -0.382* | 0.436*** | -0.146 | -0.393** |
|  | (0.14) | (0.21) | (0.21) | (0.15) | (0.21) | (0.20) | (0.15) | (0.21) | (0.20) |
| Municipality size 5 | 0.330*** | -0.371** | 0.000 | 0.370*** | -0.358* | -0.032 | 0.406*** | -0.376** | -0.040 |
|  | (0.13) | (0.19) | (0.17) | (0.13) | (0.19) | (0.16) | (0.13) | (0.18) | (0.16) |
| Municipality size 6 | 0.423*** | -0.246 | 0.069 | 0.503*** | -0.133 | 0.083 | 0.521*** | -0.264 | 0.050 |
|  | (0.13) | (0.20) | (0.17) | (0.13) | (0.20) | (0.17) | (0.13) | (0.19) | (0.17) |
| Municipality size 7 | 0.429*** | 0.099 | 0.119 | 0.466*** | 0.072 | 0.120 | 0.495*** | -0.024 | 0.109 |
|  | (0.15) | (0.23) | (0.20) | (0.15) | (0.23) | (0.19) | (0.15) | (0.23) | (0.19) |
| Gross income quintile 2 | -0.122 | -0.276* | -0.254* | -0.100 | -0.267* | -0.178 | -0.098 | -0.334** | -0.122 |
|  | (0.10) | (0.16) | (0.14) | (0.10) | (0.15) | (0.14) | (0.10) | (0.15) | (0.14) |
| Gross income quintile 3 | -0.304*** | -0.520*** | -0.331** | -0.336*** | -0.550*** | -0.231 | -0.253** | -0.493*** | -0.160 |
|  | (0.10) | (0.17) | (0.15) | (0.10) | (0.17) | (0.15) | (0.10) | (0.17) | (0.14) |
| Gross income quintile 4 | -0.541*** | -0.594*** | -0.292* | -0.533*** | -0.703*** | -0.225 | -0.503*** | -0.733*** | -0.184 |
|  | (0.11) | (0.20) | (0.16) | (0.12) | (0.20) | (0.16) | (0.12) | (0.19) | (0.16) |
| Gross income quintile 5 | -0.678*** | -0.564** | -0.062 | -0.806*** | -0.677*** | 0.010 | -0.737*** | -0.776*** | 0.049 |
|  | (0.14) | (0.24) | (0.19) | (0.14) | (0.24) | (0.18) | (0.14) | (0.24) | (0.18) |
| Home ownership | -0.096 | -0.311 | 0.495*** | -0.171 | -0.339 | 0.375** | -0.122 | -0.301 | 0.341** |
|  | (0.11) | (0.21) | (0.16) | (0.11) | (0.22) | (0.16) | (0.11) | (0.21) | (0.15) |
| Value of the house | -0.000* | -0.000 | -0.000*** | -0.000 | -0.000 | -0.000*** | -0.000* | -0.000 | -0.000*** |
|  | (0.00) | (0.00) | (0.00) | (0.00) | (0.00) | (0.00) | (0.00) | (0.00) | (0.00) |
| Financial wealth quintile 2 | 0.133 | 0.250 | -0.111 | 0.113 | 0.160 | -0.049 | 0.112 | 0.116 | -0.024 |
|  | (0.10) | (0.17) | (0.14) | (0.10) | (0.17) | (0.13) | (0.10) | (0.16) | (0.13) |
| Financial wealth quintile 3 | 0.094 | 0.097 | -0.247* | 0.100 | 0.108 | -0.206 | 0.095 | 0.030 | -0.216 |
|  | (0.10) | (0.18) | (0.14) | (0.10) | (0.17) | (0.14) | (0.10) | (0.17) | (0.14) |
| Financial wealth quintile 4 | 0.013 | 0.226 | 0.429*** | -0.032 | 0.234 | 0.467*** | -0.084 | 0.195 | 0.429*** |
|  | (0.11) | (0.19) | (0.14) | (0.11) | (0.19) | (0.14) | (0.11) | (0.18) | (0.13) |
| Financial wealth quintile 5 | 0.160 | 0.344 | -2.201*** | 0.198* | 0.387* | -2.074*** | 0.145 | 0.355 | -2.000*** |
|  | (0.12) | (0.22) | (0.24) | (0.12) | (0.22) | (0.23) | (0.12) | (0.22) | (0.22) |
| Accessibility home: 0/3 stars | 0.493** | 0.169 | 0.384 | 0.359* | 0.060 | 0.329 | 0.229 | -0.161 | 0.318 |
|  | (0.21) | (0.28) | (0.29) | (0.21) | (0.28) | (0.28) | (0.21) | (0.27) | (0.28) |
| Accessibility home: 2 stars | 0.372* | 0.027 | 0.426 | 0.290 | 0.098 | 0.367 | 0.235 | -0.140 | 0.411 |
|  | (0.20) | (0.26) | (0.28) | (0.20) | (0.27) | (0.27) | (0.20) | (0.25) | (0.27) |
| Accessibility home: 3 stars | 0.481** | 0.064 | 0.355 | 0.366* | 0.093 | 0.314 | 0.262 | -0.073 | 0.329 |
|  | (0.20) | (0.25) | (0.28) | (0.20) | (0.26) | (0.27) | (0.20) | (0.25) | (0.27) |
| Constant | -7.560*** | -11.214*** | -9.834*** | -7.684*** | -11.003*** | -9.494*** | -7.429*** | -10.362*** | -9.365*** |
|  | (0.39) | (0.68) | (0.55) | (0.39) | (0.68) | (0.53) | (0.39) | (0.66) | (0.52) |
|  |  |  |  |  |  |  |  |  |  |
| Pseudo R squared | 0.159 | 0.159 | 0.159 | 0.163 | 0.163 | 0.163 | 0.161 | 0.161 | 0.161 |
| Log likelihood | -6632 | -6632 | -6632 | -6632 | -6632 | -6632 | -6720 | -6720 | -6720 |

Regression coefficients for the sample fracture of femur at *T* weeks after the condition took place. *** p<0.01, ** p<0.05, * p<0.1.

|  | T=40 | T=40 | T=40 | T=44 | T=44 | T=44 | T=48 | T=48 | T=48 |
| --- | --- | --- | --- | --- | --- | --- | --- | --- | --- |
|  | Home care | Institutional care | Being deceased | Home care | Institutional care | Being deceased | Home care | Institutional care | Being deceased |
| Healthcare costs quintile 2 | 0.207* | 0.056 | -0.200 | 0.172 | 0.067 | -0.135 | 0.214** | 0.083 | -0.111 |
|  | (0.11) | (0.19) | (0.15) | (0.11) | (0.19) | (0.15) | (0.11) | (0.19) | (0.15) |
| Healthcare costs quintile 3 | 0.406*** | 0.229 | -0.141 | 0.425*** | 0.253 | -0.115 | 0.447*** | 0.221 | -0.092 |
|  | (0.11) | (0.18) | (0.15) | (0.10) | (0.18) | (0.15) | (0.11) | (0.18) | (0.14) |
| Healthcare costs quintile 4 | 0.560*** | 0.279 | 0.462*** | 0.528*** | 0.219 | 0.479*** | 0.621*** | 0.255 | 0.536*** |
|  | (0.11) | (0.19) | (0.14) | (0.11) | (0.19) | (0.13) | (0.11) | (0.19) | (0.13) |
| Healthcare costs quintile 5 | 0.919*** | 1.228*** | 1.003*** | 0.876*** | 1.224*** | 1.014*** | 0.971*** | 1.201*** | 1.086*** |
|  | (0.11) | (0.17) | (0.13) | (0.11) | (0.17) | (0.13) | (0.11) | (0.17) | (0.13) |
| Eligibility for long-term care t-14 | 0.198* | 1.177*** | 1.330*** | 0.239** | 1.204*** | 1.324*** | 0.261** | 1.227*** | 1.325*** |
|  | (0.12) | (0.14) | (0.12) | (0.12) | (0.14) | (0.11) | (0.12) | (0.14) | (0.11) |
| Man | -0.146** | 0.104 | 0.753*** | -0.170** | 0.132 | 0.739*** | -0.149** | 0.172 | 0.758*** |
|  | (0.07) | (0.12) | (0.09) | (0.07) | (0.12) | (0.09) | (0.07) | (0.12) | (0.09) |
| Partner | -0.379*** | -0.302** | -0.189* | -0.312*** | -0.306** | -0.155 | -0.287*** | -0.278** | -0.152 |
|  | (0.07) | (0.13) | (0.10) | (0.07) | (0.13) | (0.10) | (0.08) | (0.13) | (0.10) |
| Children living in the household | -0.003 | 0.108 | 0.144 | -0.011 | 0.049 | 0.162 | -0.077 | 0.049 | 0.134 |
|  | (0.13) | (0.24) | (0.16) | (0.13) | (0.24) | (0.15) | (0.14) | (0.24) | (0.15) |
| Number of children | 0.047** | 0.001 | -0.029 | 0.030 | -0.004 | -0.036 | 0.027 | -0.001 | -0.032 |
|  | (0.02) | (0.04) | (0.03) | (0.02) | (0.04) | (0.03) | (0.02) | (0.04) | (0.03) |
| Having children | -0.346*** | -0.602*** | -0.352** | -0.260** | -0.584*** | -0.317** | -0.253** | -0.598*** | -0.324** |
|  | (0.11) | (0.16) | (0.14) | (0.11) | (0.17) | (0.14) | (0.11) | (0.16) | (0.14) |
| First generation immigrant | 0.079 | -0.454* | -0.286 | 0.134 | -0.315 | -0.293 | 0.134 | -0.309 | -0.227 |
|  | (0.14) | (0.24) | (0.19) | (0.14) | (0.23) | (0.18) | (0.14) | (0.23) | (0.18) |
| Second generation immigrant | 0.120 | -0.053 | -0.013 | 0.148 | -0.036 | 0.081 | 0.130 | 0.043 | 0.074 |
|  | (0.14) | (0.25) | (0.19) | (0.14) | (0.25) | (0.18) | (0.14) | (0.24) | (0.18) |
| Age | 0.082*** | 0.116*** | 0.096*** | 0.081*** | 0.118*** | 0.094*** | 0.083*** | 0.118*** | 0.095*** |
|  | (0.00) | (0.01) | (0.01) | (0.00) | (0.01) | (0.01) | (0.00) | (0.01) | (0.01) |
| Urbanity category 2 | -0.044 | -0.160 | -0.189 | -0.042 | -0.162 | -0.241* | 0.031 | -0.133 | -0.239* |
|  | (0.10) | (0.15) | (0.13) | (0.10) | (0.15) | (0.13) | (0.10) | (0.15) | (0.13) |
| Urbanity category 3 | 0.042 | -0.426** | -0.188 | 0.041 | -0.337* | -0.252* | 0.104 | -0.291 | -0.248* |
|  | (0.12) | (0.19) | (0.15) | (0.12) | (0.19) | (0.15) | (0.12) | (0.19) | (0.15) |
| Urbanity category 4 | 0.066 | -0.587*** | -0.139 | 0.021 | -0.733*** | -0.171 | 0.112 | -0.613*** | -0.174 |
|  | (0.13) | (0.22) | (0.17) | (0.13) | (0.23) | (0.16) | (0.13) | (0.22) | (0.16) |
| Urbanity category 5 | 0.230* | -0.410* | -0.116 | 0.198 | -0.354 | -0.172 | 0.243* | -0.388* | -0.182 |
|  | (0.13) | (0.23) | (0.17) | (0.13) | (0.23) | (0.17) | (0.13) | (0.23) | (0.17) |
| Municipality size 2 | -0.352 | -0.285 | -0.109 | -0.492 | -0.242 | -0.015 | -0.424 | -0.237 | -0.034 |
|  | (0.29) | (0.37) | (0.30) | (0.31) | (0.38) | (0.28) | (0.30) | (0.38) | (0.28) |
| Municipality size 3 | 0.231 | -0.149 | -0.122 | 0.150 | -0.154 | -0.169 | 0.112 | -0.154 | -0.210 |
|  | (0.16) | (0.22) | (0.19) | (0.16) | (0.22) | (0.19) | (0.16) | (0.22) | (0.19) |
| Municipality size 4 | 0.472*** | 0.129 | -0.385* | 0.409*** | 0.117 | -0.418** | 0.416*** | 0.169 | -0.411** |
|  | (0.15) | (0.20) | (0.20) | (0.15) | (0.20) | (0.19) | (0.15) | (0.20) | (0.19) |
| Municipality size 5 | 0.423*** | -0.397** | -0.066 | 0.411*** | -0.381** | -0.084 | 0.331** | -0.368** | -0.073 |
|  | (0.13) | (0.18) | (0.16) | (0.13) | (0.19) | (0.15) | (0.13) | (0.19) | (0.15) |
| Municipality size 6 | 0.528*** | -0.280 | 0.042 | 0.503*** | -0.263 | 0.026 | 0.438*** | -0.253 | 0.026 |
|  | (0.14) | (0.20) | (0.16) | (0.14) | (0.20) | (0.16) | (0.14) | (0.20) | (0.16) |
| Municipality size 7 | 0.481*** | 0.021 | 0.077 | 0.445*** | 0.010 | 0.025 | 0.452*** | 0.054 | 0.101 |
|  | (0.15) | (0.23) | (0.19) | (0.15) | (0.23) | (0.18) | (0.15) | (0.23) | (0.18) |
| Gross income quintile 2 | -0.115 | -0.296* | -0.143 | -0.073 | -0.227 | -0.154 | -0.072 | -0.185 | -0.131 |
|  | (0.10) | (0.15) | (0.13) | (0.10) | (0.15) | (0.13) | (0.10) | (0.15) | (0.13) |
| Gross income quintile 3 | -0.282*** | -0.445*** | -0.172 | -0.284*** | -0.502*** | -0.145 | -0.291*** | -0.513*** | -0.130 |
|  | (0.11) | (0.17) | (0.14) | (0.11) | (0.17) | (0.14) | (0.11) | (0.17) | (0.14) |
| Gross income quintile 4 | -0.435*** | -0.721*** | -0.198 | -0.465*** | -0.751*** | -0.200 | -0.443*** | -0.803*** | -0.197 |
|  | (0.12) | (0.20) | (0.15) | (0.12) | (0.20) | (0.15) | (0.12) | (0.20) | (0.15) |
| Gross income quintile 5 | -0.626*** | -0.791*** | 0.018 | -0.552*** | -0.766*** | 0.040 | -0.586*** | -0.825*** | 0.047 |
|  | (0.14) | (0.24) | (0.18) | (0.14) | (0.24) | (0.17) | (0.14) | (0.24) | (0.17) |
| Home ownership | -0.108 | -0.290 | 0.355** | -0.072 | -0.490** | 0.367** | -0.030 | -0.450** | 0.335** |
|  | (0.12) | (0.21) | (0.15) | (0.12) | (0.21) | (0.15) | (0.12) | (0.22) | (0.14) |
| Value of the house | -0.000** | -0.000 | -0.000*** | -0.000** | -0.000 | -0.000*** | -0.000*** | -0.000 | -0.000*** |
|  | (0.00) | (0.00) | (0.00) | (0.00) | (0.00) | (0.00) | (0.00) | (0.00) | (0.00) |
| Financial wealth quintile 2 | 0.066 | 0.208 | -0.030 | 0.083 | 0.145 | -0.007 | 0.115 | 0.158 | 0.014 |
|  | (0.10) | (0.16) | (0.13) | (0.10) | (0.17) | (0.13) | (0.10) | (0.16) | (0.12) |
| Financial wealth quintile 3 | 0.067 | 0.034 | -0.213 | 0.034 | 0.046 | -0.184 | 0.096 | 0.012 | -0.178 |
|  | (0.10) | (0.18) | (0.14) | (0.10) | (0.18) | (0.13) | (0.10) | (0.17) | (0.13) |
| Financial wealth quintile 4 | -0.071 | 0.286 | 0.435*** | -0.069 | 0.257 | 0.439*** | -0.056 | 0.235 | 0.432*** |
|  | (0.11) | (0.18) | (0.13) | (0.11) | (0.19) | (0.13) | (0.12) | (0.19) | (0.13) |
| Financial wealth quintile 5 | 0.098 | 0.392* | -1.942*** | 0.073 | 0.502** | -1.775*** | 0.134 | 0.527** | -1.694*** |
|  | (0.12) | (0.22) | (0.21) | (0.12) | (0.22) | (0.20) | (0.12) | (0.22) | (0.19) |
| Accessibility home: 0/3 stars | 0.044 | 0.012 | 0.252 | 0.161 | 0.018 | 0.363 | 0.193 | 0.016 | 0.297 |
|  | (0.20) | (0.28) | (0.27) | (0.21) | (0.28) | (0.27) | (0.21) | (0.28) | (0.27) |
| Accessibility home: 2 stars | -0.015 | -0.072 | 0.298 | 0.087 | -0.037 | 0.391 | 0.104 | -0.032 | 0.328 |
|  | (0.19) | (0.27) | (0.26) | (0.20) | (0.27) | (0.26) | (0.20) | (0.27) | (0.25) |
| Accessibility home: 3 stars | 0.050 | 0.007 | 0.231 | 0.189 | 0.071 | 0.369 | 0.198 | 0.074 | 0.331 |
|  | (0.19) | (0.26) | (0.26) | (0.19) | (0.26) | (0.26) | (0.20) | (0.26) | (0.25) |
| Constant | -7.556*** | -10.551*** | -9.398*** | -7.649*** | -10.801*** | -9.308*** | -7.974*** | -10.794*** | -9.299*** |
|  | (0.39) | (0.67) | (0.51) | (0.40) | (0.68) | (0.50) | (0.40) | (0.67) | (0.49) |
|  |  |  |  |  |  |  |  |  |  |
| Pseudo R squared | 0.162 | 0.162 | 0.162 | 0.159 | 0.159 | 0.159 | 0.162 | 0.162 | 0.162 |
| Log likelihood | -6735 | -6735 | -6735 | -6793 | -6793 | -6793 | -6815 | -6815 | -6815 |

Regression coefficients for the sample fracture of femur at *T* weeks after the condition took place. *** p<0.01, ** p<0.05, * p<0.1.

|  | T=52 | T=52 | T=52 |
| --- | --- | --- | --- |
|  | Home care | Institutional care | Being deceased |
| Healthcare costs quintile 2 | 0.219** | 0.125 | -0.063 |
|  | (0.11) | (0.19) | (0.14) |
| Healthcare costs quintile 3 | 0.413*** | 0.320* | -0.032 |
|  | (0.11) | (0.18) | (0.14) |
| Healthcare costs quintile 4 | 0.564*** | 0.297 | 0.553*** |
|  | (0.11) | (0.19) | (0.13) |
| Healthcare costs quintile 5 | 0.923*** | 1.266*** | 1.129*** |
|  | (0.11) | (0.17) | (0.13) |
| Eligibility for long-term care t-14 | 0.220* | 1.272*** | 1.298*** |
|  | (0.12) | (0.14) | (0.11) |
| Man | -0.121* | 0.148 | 0.713*** |
|  | (0.07) | (0.12) | (0.08) |
| Partner | -0.364*** | -0.251* | -0.130 |
|  | (0.08) | (0.13) | (0.09) |
| Children living in the household | 0.054 | 0.003 | 0.173 |
|  | (0.13) | (0.25) | (0.15) |
| Number of children | 0.033 | -0.005 | -0.033 |
|  | (0.02) | (0.04) | (0.03) |
| Having children | -0.272** | -0.623*** | -0.339** |
|  | (0.11) | (0.16) | (0.13) |
| First generation immigrant | 0.178 | -0.283 | -0.167 |
|  | (0.14) | (0.23) | (0.18) |
| Second generation immigrant | 0.178 | 0.002 | 0.137 |
|  | (0.14) | (0.25) | (0.18) |
| Age | 0.085*** | 0.118*** | 0.095*** |
|  | (0.00) | (0.01) | (0.01) |
| Urbanity category 2 | 0.032 | -0.034 | -0.267** |
|  | (0.11) | (0.15) | (0.12) |
| Urbanity category 3 | 0.135 | -0.321* | -0.276* |
|  | (0.12) | (0.19) | (0.14) |
| Urbanity category 4 | 0.193 | -0.587*** | -0.184 |
|  | (0.13) | (0.22) | (0.16) |
| Urbanity category 5 | 0.266** | -0.348 | -0.205 |
|  | (0.13) | (0.23) | (0.17) |
| Municipality size 2 | -0.213 | -0.147 | 0.103 |
|  | (0.29) | (0.38) | (0.28) |
| Municipality size 3 | 0.227 | -0.083 | -0.150 |
|  | (0.16) | (0.22) | (0.18) |
| Municipality size 4 | 0.408*** | 0.186 | -0.386** |
|  | (0.15) | (0.20) | (0.19) |
| Municipality size 5 | 0.393*** | -0.382** | -0.035 |
|  | (0.13) | (0.19) | (0.15) |
| Municipality size 6 | 0.443*** | -0.211 | 0.053 |
|  | (0.14) | (0.20) | (0.16) |
| Municipality size 7 | 0.442*** | 0.164 | 0.140 |
|  | (0.15) | (0.23) | (0.18) |
| Gross income quintile 2 | -0.097 | -0.221 | -0.152 |
|  | (0.10) | (0.15) | (0.13) |
| Gross income quintile 3 | -0.327*** | -0.505*** | -0.106 |
|  | (0.11) | (0.17) | (0.13) |
| Gross income quintile 4 | -0.499*** | -0.731*** | -0.170 |
|  | (0.12) | (0.20) | (0.15) |
| Gross income quintile 5 | -0.673*** | -0.752*** | 0.061 |
|  | (0.14) | (0.24) | (0.17) |
| Home ownership | -0.039 | -0.452** | 0.358** |
|  | (0.12) | (0.21) | (0.14) |
| Value of the house | -0.000** | -0.000 | -0.000*** |
|  | (0.00) | (0.00) | (0.00) |
| Financial wealth quintile 2 | 0.081 | 0.106 | -0.057 |
|  | (0.10) | (0.16) | (0.12) |
| Financial wealth quintile 3 | 0.087 | -0.013 | -0.229* |
|  | (0.10) | (0.17) | (0.13) |
| Financial wealth quintile 4 | -0.101 | 0.086 | 0.389*** |
|  | (0.12) | (0.19) | (0.12) |
| Financial wealth quintile 5 | 0.138 | 0.378* | -1.674*** |
|  | (0.12) | (0.22) | (0.19) |
| Accessibility home: 0/3 stars | 0.229 | -0.057 | 0.402 |
|  | (0.21) | (0.27) | (0.26) |
| Accessibility home: 2 stars | 0.142 | -0.130 | 0.382 |
|  | (0.20) | (0.26) | (0.25) |
| Accessibility home: 3 stars | 0.217 | -0.037 | 0.365 |
|  | (0.20) | (0.25) | (0.25) |
| Constant | -8.062*** | -10.767*** | -9.294*** |
|  | (0.41) | (0.67) | (0.49) |
|  |  |  |  |
| Pseudo R squared | 0.163 | 0.163 | 0.163 |
| Log likelihood | -6847 | -6847 | -6847 |

Regression coefficients for the sample fracture of femur at *T* weeks after the condition took place. *** p<0.01, ** p<0.05, * p<0.1.

A.2 Stroke (N=7,884)

|  | T=4 | T=4 | T=4 | T=8 | T=8 | T=8 | T=12 | T=12 | T=12 |
| --- | --- | --- | --- | --- | --- | --- | --- | --- | --- |
|  | Home care | Institutional care | Being deceased | Home care | Institutional care | Being deceased | Home care | Institutional care | Being deceased |
| Healthcare costs quintile 2 | -0.047 | 0.200 | -0.120 | 0.024 | -0.043 | -0.112 | 0.033 | 0.076 | -0.085 |
|  | (0.09) | (0.20) | (0.08) | (0.08) | (0.16) | (0.07) | (0.08) | (0.14) | (0.07) |
| Healthcare costs quintile 3 | 0.113 | 0.208 | -0.069 | 0.136* | -0.121 | -0.051 | 0.090 | -0.211 | -0.058 |
|  | (0.09) | (0.20) | (0.08) | (0.08) | (0.16) | (0.07) | (0.08) | (0.15) | (0.07) |
| Healthcare costs quintile 4 | 0.144* | 0.390** | 0.065 | 0.243*** | 0.045 | 0.096 | 0.221*** | -0.085 | 0.081 |
|  | (0.09) | (0.19) | (0.08) | (0.08) | (0.16) | (0.07) | (0.08) | (0.14) | (0.07) |
| Healthcare costs quintile 5 | 0.476*** | 0.815*** | 0.138* | 0.640*** | 0.870*** | 0.237*** | 0.593*** | 1.011*** | 0.294*** |
|  | (0.08) | (0.19) | (0.08) | (0.08) | (0.14) | (0.07) | (0.07) | (0.12) | (0.07) |
| Eligibility for long-term care t-14 | 0.569*** | 0.768*** | 0.599*** | 0.436*** | 0.842*** | 0.618*** | 0.419*** | 0.678*** | 0.668*** |
|  | (0.09) | (0.16) | (0.09) | (0.09) | (0.14) | (0.09) | (0.09) | (0.13) | (0.08) |
| Man | -0.235*** | -0.348*** | 0.087* | -0.223*** | -0.267*** | 0.107** | -0.209*** | -0.270*** | 0.094** |
|  | (0.05) | (0.12) | (0.05) | (0.05) | (0.10) | (0.05) | (0.05) | (0.08) | (0.05) |
| Partner | -0.328*** | -0.374*** | -0.099* | -0.474*** | -0.505*** | -0.101* | -0.518*** | -0.490*** | -0.115** |
|  | (0.06) | (0.13) | (0.06) | (0.06) | (0.11) | (0.05) | (0.05) | (0.09) | (0.05) |
| Children living in the household | -0.095 | 0.315 | 0.128 | -0.012 | 0.274 | 0.123 | 0.030 | 0.277* | 0.133* |
|  | (0.11) | (0.21) | (0.09) | (0.10) | (0.17) | (0.08) | (0.09) | (0.16) | (0.08) |
| Number of children | 0.059*** | -0.070* | -0.037* | 0.047*** | 0.006 | -0.026 | 0.036** | 0.002 | -0.027 |
|  | (0.02) | (0.04) | (0.02) | (0.02) | (0.03) | (0.02) | (0.02) | (0.03) | (0.02) |
| Having children | -0.144 | -0.073 | -0.086 | -0.134 | -0.276* | -0.102 | -0.121 | -0.237* | -0.134* |
|  | (0.09) | (0.19) | (0.09) | (0.09) | (0.15) | (0.08) | (0.08) | (0.14) | (0.08) |
| First generation immigrant | -0.004 | 0.046 | 0.023 | -0.156* | 0.081 | 0.021 | -0.105 | 0.035 | 0.001 |
|  | (0.10) | (0.19) | (0.09) | (0.09) | (0.16) | (0.08) | (0.09) | (0.14) | (0.08) |
| Second generation immigrant | -0.134 | -0.374 | -0.071 | -0.160 | -0.030 | -0.090 | -0.114 | -0.141 | -0.059 |
|  | (0.12) | (0.28) | (0.11) | (0.11) | (0.20) | (0.10) | (0.10) | (0.18) | (0.10) |
| Age | 0.071*** | 0.097*** | 0.055*** | 0.074*** | 0.096*** | 0.061*** | 0.079*** | 0.110*** | 0.064*** |
|  | (0.00) | (0.01) | (0.00) | (0.00) | (0.01) | (0.00) | (0.00) | (0.01) | (0.00) |
| Urbanity category 2 | 0.009 | 0.013 | -0.084 | -0.073 | 0.147 | -0.051 | -0.023 | 0.030 | -0.029 |
|  | (0.09) | (0.18) | (0.08) | (0.08) | (0.15) | (0.07) | (0.08) | (0.13) | (0.07) |
| Urbanity category 3 | -0.102 | -0.049 | -0.117 | -0.002 | 0.149 | -0.069 | -0.002 | -0.223 | -0.068 |
|  | (0.10) | (0.21) | (0.09) | (0.09) | (0.18) | (0.08) | (0.09) | (0.15) | (0.08) |
| Urbanity category 4 | 0.027 | 0.195 | -0.136 | 0.081 | 0.263 | -0.085 | 0.096 | -0.005 | -0.063 |
|  | (0.11) | (0.22) | (0.10) | (0.10) | (0.19) | (0.09) | (0.09) | (0.16) | (0.09) |
| Urbanity category 5 | 0.214** | -0.016 | -0.118 | 0.216** | 0.149 | -0.082 | 0.279*** | -0.062 | -0.065 |
|  | (0.11) | (0.24) | (0.10) | (0.10) | (0.20) | (0.10) | (0.10) | (0.17) | (0.09) |
| Municipality size 2 | -0.025 | -0.626 | 0.383** | -0.193 | -0.231 | 0.314** | -0.280 | -0.441 | 0.332** |
|  | (0.20) | (0.48) | (0.16) | (0.19) | (0.34) | (0.15) | (0.19) | (0.32) | (0.15) |
| Municipality size 3 | 0.302** | -0.505* | 0.217* | 0.146 | -0.393 | 0.234** | 0.198* | -0.028 | 0.210* |
|  | (0.13) | (0.30) | (0.12) | (0.12) | (0.24) | (0.11) | (0.12) | (0.19) | (0.11) |
| Municipality size 4 | 0.311** | -0.154 | 0.135 | 0.281** | -0.245 | 0.158 | 0.345*** | -0.113 | 0.143 |
|  | (0.12) | (0.25) | (0.12) | (0.11) | (0.21) | (0.11) | (0.11) | (0.18) | (0.10) |
| Municipality size 5 | 0.199* | -0.340 | 0.222** | 0.166 | -0.198 | 0.251*** | 0.282*** | -0.067 | 0.260*** |
|  | (0.11) | (0.22) | (0.10) | (0.10) | (0.18) | (0.10) | (0.10) | (0.16) | (0.09) |
| Municipality size 6 | 0.295*** | 0.003 | 0.229** | 0.220** | -0.062 | 0.235** | 0.262** | 0.089 | 0.220** |
|  | (0.11) | (0.22) | (0.11) | (0.11) | (0.19) | (0.10) | (0.10) | (0.16) | (0.10) |
| Municipality size 7 | 0.312** | -0.071 | 0.181 | 0.190 | -0.044 | 0.187* | 0.169 | 0.116 | 0.189* |
|  | (0.13) | (0.25) | (0.12) | (0.12) | (0.21) | (0.11) | (0.11) | (0.18) | (0.11) |
| Gross income quintile 2 | 0.026 | -0.215 | 0.082 | -0.051 | -0.259* | 0.037 | -0.162** | -0.314*** | -0.046 |
|  | (0.07) | (0.16) | (0.08) | (0.07) | (0.13) | (0.07) | (0.07) | (0.11) | (0.07) |
| Gross income quintile 3 | -0.160* | -0.452** | 0.090 | -0.333*** | -0.342** | 0.035 | -0.441*** | -0.472*** | -0.053 |
|  | (0.08) | (0.18) | (0.08) | (0.08) | (0.15) | (0.08) | (0.07) | (0.13) | (0.07) |
| Gross income quintile 4 | -0.347*** | -0.398** | 0.089 | -0.471*** | -0.413** | 0.027 | -0.629*** | -0.589*** | -0.015 |
|  | (0.09) | (0.19) | (0.09) | (0.09) | (0.16) | (0.08) | (0.09) | (0.14) | (0.08) |
| Gross income quintile 5 | -0.438*** | -0.325 | 0.035 | -0.465*** | -0.250 | 0.043 | -0.544*** | -0.411** | 0.002 |
|  | (0.12) | (0.23) | (0.10) | (0.11) | (0.19) | (0.09) | (0.10) | (0.17) | (0.09) |
| Home ownership | -0.022 | 0.242 | -0.056 | 0.039 | 0.374** | -0.131* | 0.111 | 0.301* | -0.097 |
|  | (0.09) | (0.21) | (0.08) | (0.09) | (0.18) | (0.08) | (0.08) | (0.16) | (0.07) |
| Value of the house | -0.000** | -0.000*** | -0.000 | -0.000*** | -0.000*** | -0.000* | -0.000*** | -0.000*** | -0.000** |
|  | (0.00) | (0.00) | (0.00) | (0.00) | (0.00) | (0.00) | (0.00) | (0.00) | (0.00) |
| Financial wealth quintile 2 | 0.016 | -0.401** | 0.121 | -0.031 | -0.259* | 0.099 | -0.102 | -0.167 | 0.094 |
|  | (0.08) | (0.18) | (0.08) | (0.08) | (0.14) | (0.07) | (0.07) | (0.13) | (0.07) |
| Financial wealth quintile 3 | -0.058 | -0.422** | -0.089 | -0.078 | -0.379** | -0.046 | -0.081 | -0.122 | -0.038 |
|  | (0.09) | (0.18) | (0.08) | (0.08) | (0.15) | (0.08) | (0.07) | (0.13) | (0.07) |
| Financial wealth quintile 4 | -0.194** | -0.148 | 0.093 | -0.199** | -0.224 | 0.058 | -0.239*** | -0.018 | 0.044 |
|  | (0.09) | (0.18) | (0.08) | (0.09) | (0.16) | (0.08) | (0.08) | (0.14) | (0.08) |
| Financial wealth quintile 5 | 0.096 | 0.083 | 0.224** | 0.052 | 0.003 | 0.230*** | -0.018 | 0.144 | 0.232*** |
|  | (0.10) | (0.21) | (0.09) | (0.09) | (0.17) | (0.09) | (0.09) | (0.15) | (0.08) |
| Accessibility home: 0/3 stars | -0.203 | -0.632** | -0.157 | 0.013 | -0.403* | -0.122 | -0.104 | -0.506** | -0.097 |
|  | (0.17) | (0.27) | (0.16) | (0.16) | (0.24) | (0.15) | (0.15) | (0.20) | (0.15) |
| Accessibility home: 2 stars | -0.168 | -0.643*** | -0.006 | 0.069 | -0.506** | -0.016 | -0.017 | -0.628*** | -0.009 |
|  | (0.15) | (0.25) | (0.15) | (0.15) | (0.22) | (0.14) | (0.14) | (0.19) | (0.14) |
| Accessibility home: 3 stars | -0.069 | -0.742*** | -0.070 | 0.170 | -0.500** | -0.032 | 0.068 | -0.630*** | -0.050 |
|  | (0.15) | (0.25) | (0.15) | (0.15) | (0.22) | (0.14) | (0.14) | (0.19) | (0.14) |
| Constant | -7.551*** | -9.997*** | -6.466*** | -7.604*** | -9.612*** | -6.727*** | -7.581*** | -10.260*** | -6.740*** |
|  | (0.32) | (0.65) | (0.29) | (0.30) | (0.54) | (0.27) | (0.29) | (0.48) | (0.26) |
|  |  |  |  |  |  |  |  |  |  |
| Pseudo R squared | 0.0693 | 0.0693 | 0.0693 | 0.0795 | 0.0795 | 0.0795 | 0.0899 | 0.0899 | 0.0899 |
| Log likelihood | -14241 | -14241 | -14241 | -16211 | -16211 | -16211 | -17582 | -17582 | -17582 |

Regression coefficients for the stroke sample at *T* weeks after the condition took place. *** p<0.01, ** p<0.05, * p<0.1.

|  | T=16 | T=16 | T=16 | T=20 | T=20 | T=20 | T=24 | T=24 | T=24 |
| --- | --- | --- | --- | --- | --- | --- | --- | --- | --- |
|  | Home care | Institutional care | Being deceased | Home care | Institutional care | Being deceased | Home care | Institutional care | Being deceased |
| Healthcare costs quintile 2 | -0.041 | -0.006 | -0.076 | -0.046 | -0.145 | -0.075 | -0.066 | -0.222** | -0.089 |
|  | (0.08) | (0.12) | (0.07) | (0.07) | (0.11) | (0.07) | (0.07) | (0.10) | (0.07) |
| Healthcare costs quintile 3 | 0.101 | -0.241* | -0.040 | 0.081 | -0.441*** | -0.058 | 0.038 | -0.439*** | -0.062 |
|  | (0.07) | (0.13) | (0.07) | (0.07) | (0.11) | (0.07) | (0.07) | (0.11) | (0.07) |
| Healthcare costs quintile 4 | 0.163** | -0.213* | 0.073 | 0.192*** | -0.356*** | 0.095 | 0.176** | -0.408*** | 0.070 |
|  | (0.07) | (0.13) | (0.07) | (0.07) | (0.11) | (0.07) | (0.07) | (0.11) | (0.07) |
| Healthcare costs quintile 5 | 0.564*** | 0.856*** | 0.372*** | 0.560*** | 0.708*** | 0.402*** | 0.521*** | 0.632*** | 0.423*** |
|  | (0.07) | (0.11) | (0.07) | (0.07) | (0.10) | (0.07) | (0.07) | (0.09) | (0.06) |
| Eligibility for long-term care t-14 | 0.321*** | 0.677*** | 0.656*** | 0.333*** | 0.559*** | 0.667*** | 0.412*** | 0.637*** | 0.688*** |
|  | (0.09) | (0.12) | (0.08) | (0.09) | (0.11) | (0.08) | (0.09) | (0.11) | (0.08) |
| Man | -0.213*** | -0.228*** | 0.093** | -0.198*** | -0.267*** | 0.099** | -0.200*** | -0.248*** | 0.109** |
|  | (0.05) | (0.08) | (0.04) | (0.04) | (0.07) | (0.04) | (0.04) | (0.07) | (0.04) |
| Partner | -0.534*** | -0.581*** | -0.126** | -0.532*** | -0.638*** | -0.157*** | -0.554*** | -0.633*** | -0.169*** |
|  | (0.05) | (0.08) | (0.05) | (0.05) | (0.08) | (0.05) | (0.05) | (0.07) | (0.05) |
| Children living in the household | 0.063 | 0.326** | 0.152* | 0.111 | 0.282** | 0.153** | 0.095 | 0.303** | 0.140* |
|  | (0.09) | (0.14) | (0.08) | (0.09) | (0.13) | (0.08) | (0.09) | (0.12) | (0.08) |
| Number of children | 0.039** | -0.006 | -0.026 | 0.036** | -0.004 | -0.028 | 0.021 | -0.007 | -0.032* |
|  | (0.02) | (0.03) | (0.02) | (0.02) | (0.02) | (0.02) | (0.02) | (0.02) | (0.02) |
| Having children | -0.186** | -0.305** | -0.132* | -0.199** | -0.280** | -0.154** | -0.160** | -0.297*** | -0.169** |
|  | (0.08) | (0.12) | (0.08) | (0.08) | (0.11) | (0.08) | (0.08) | (0.11) | (0.07) |
| First generation immigrant | -0.034 | 0.127 | -0.022 | -0.055 | 0.153 | -0.017 | -0.084 | 0.106 | -0.003 |
|  | (0.08) | (0.12) | (0.08) | (0.08) | (0.12) | (0.08) | (0.08) | (0.11) | (0.08) |
| Second generation immigrant | -0.120 | 0.043 | -0.052 | -0.149 | 0.093 | -0.065 | -0.134 | 0.071 | -0.089 |
|  | (0.10) | (0.15) | (0.09) | (0.10) | (0.14) | (0.09) | (0.10) | (0.13) | (0.09) |
| Age | 0.075*** | 0.106*** | 0.066*** | 0.072*** | 0.103*** | 0.069*** | 0.070*** | 0.095*** | 0.070*** |
|  | (0.00) | (0.00) | (0.00) | (0.00) | (0.00) | (0.00) | (0.00) | (0.00) | (0.00) |
| Urbanity category 2 | -0.035 | 0.028 | -0.040 | -0.003 | 0.041 | -0.054 | -0.027 | 0.041 | -0.042 |
|  | (0.07) | (0.12) | (0.07) | (0.07) | (0.11) | (0.07) | (0.07) | (0.10) | (0.07) |
| Urbanity category 3 | -0.026 | -0.086 | -0.063 | -0.067 | -0.121 | -0.060 | -0.063 | -0.135 | -0.068 |
|  | (0.08) | (0.14) | (0.08) | (0.08) | (0.13) | (0.08) | (0.08) | (0.12) | (0.08) |
| Urbanity category 4 | 0.075 | 0.011 | -0.063 | 0.072 | 0.087 | -0.073 | 0.045 | 0.070 | -0.066 |
|  | (0.09) | (0.15) | (0.09) | (0.09) | (0.14) | (0.08) | (0.09) | (0.13) | (0.08) |
| Urbanity category 5 | 0.249*** | 0.049 | -0.053 | 0.187** | 0.140 | -0.061 | 0.198** | 0.136 | -0.047 |
|  | (0.09) | (0.15) | (0.09) | (0.09) | (0.14) | (0.09) | (0.09) | (0.13) | (0.09) |
| Municipality size 2 | -0.103 | -0.515* | 0.288** | -0.180 | -0.680** | 0.231 | -0.283* | -0.405* | 0.162 |
|  | (0.17) | (0.29) | (0.14) | (0.17) | (0.28) | (0.14) | (0.17) | (0.23) | (0.14) |
| Municipality size 3 | 0.203* | -0.118 | 0.198* | 0.173 | -0.084 | 0.162 | 0.147 | -0.058 | 0.132 |
|  | (0.11) | (0.17) | (0.11) | (0.11) | (0.16) | (0.11) | (0.11) | (0.15) | (0.10) |
| Municipality size 4 | 0.301*** | -0.243 | 0.122 | 0.293*** | -0.085 | 0.113 | 0.206** | -0.119 | 0.063 |
|  | (0.10) | (0.16) | (0.10) | (0.10) | (0.15) | (0.10) | (0.10) | (0.14) | (0.10) |
| Municipality size 5 | 0.283*** | -0.060 | 0.252*** | 0.223** | -0.162 | 0.219** | 0.142 | -0.221* | 0.169** |
|  | (0.09) | (0.14) | (0.09) | (0.09) | (0.13) | (0.09) | (0.09) | (0.12) | (0.09) |
| Municipality size 6 | 0.239** | -0.056 | 0.183** | 0.204** | -0.149 | 0.157* | 0.197** | -0.190 | 0.112 |
|  | (0.10) | (0.15) | (0.09) | (0.09) | (0.13) | (0.09) | (0.09) | (0.13) | (0.09) |
| Municipality size 7 | 0.108 | -0.034 | 0.147 | 0.096 | -0.180 | 0.143 | 0.033 | -0.172 | 0.113 |
|  | (0.11) | (0.17) | (0.10) | (0.11) | (0.15) | (0.10) | (0.10) | (0.14) | (0.10) |
| Gross income quintile 2 | -0.129** | -0.283*** | -0.054 | -0.107* | -0.242** | -0.055 | -0.128** | -0.268*** | -0.074 |
|  | (0.06) | (0.10) | (0.07) | (0.06) | (0.09) | (0.07) | (0.06) | (0.09) | (0.07) |
| Gross income quintile 3 | -0.462*** | -0.495*** | -0.072 | -0.459*** | -0.400*** | -0.079 | -0.451*** | -0.358*** | -0.096 |
|  | (0.07) | (0.11) | (0.07) | (0.07) | (0.10) | (0.07) | (0.07) | (0.10) | (0.07) |
| Gross income quintile 4 | -0.674*** | -0.653*** | -0.045 | -0.632*** | -0.523*** | -0.041 | -0.604*** | -0.460*** | -0.061 |
|  | (0.08) | (0.13) | (0.08) | (0.08) | (0.12) | (0.07) | (0.08) | (0.11) | (0.07) |
| Gross income quintile 5 | -0.606*** | -0.583*** | -0.031 | -0.636*** | -0.659*** | -0.046 | -0.639*** | -0.734*** | -0.071 |
|  | (0.10) | (0.16) | (0.09) | (0.10) | (0.15) | (0.09) | (0.09) | (0.14) | (0.09) |
| Home ownership | -0.028 | 0.153 | -0.094 | -0.005 | 0.203 | -0.101 | -0.013 | 0.198 | -0.117* |
|  | (0.08) | (0.14) | (0.07) | (0.08) | (0.13) | (0.07) | (0.08) | (0.12) | (0.07) |
| Value of the house | -0.000** | -0.000*** | -0.000*** | -0.000*** | -0.000*** | -0.000** | -0.000** | -0.000*** | -0.000** |
|  | (0.00) | (0.00) | (0.00) | (0.00) | (0.00) | (0.00) | (0.00) | (0.00) | (0.00) |
| Financial wealth quintile 2 | -0.023 | -0.014 | 0.073 | -0.044 | 0.042 | 0.051 | -0.045 | 0.015 | 0.017 |
|  | (0.07) | (0.12) | (0.07) | (0.07) | (0.11) | (0.07) | (0.07) | (0.10) | (0.07) |
| Financial wealth quintile 3 | -0.079 | 0.012 | -0.064 | -0.066 | 0.051 | -0.083 | -0.031 | 0.031 | -0.076 |
|  | (0.07) | (0.12) | (0.07) | (0.07) | (0.11) | (0.07) | (0.07) | (0.10) | (0.07) |
| Financial wealth quintile 4 | -0.215*** | -0.023 | 0.028 | -0.173** | 0.070 | 0.007 | -0.137* | 0.070 | 0.008 |
|  | (0.08) | (0.13) | (0.07) | (0.08) | (0.12) | (0.07) | (0.08) | (0.11) | (0.07) |
| Financial wealth quintile 5 | 0.046 | 0.238* | 0.230*** | 0.045 | 0.294** | 0.194** | 0.043 | 0.298** | 0.184** |
|  | (0.08) | (0.14) | (0.08) | (0.08) | (0.13) | (0.08) | (0.08) | (0.12) | (0.08) |
| Accessibility home: 0/3 stars | 0.023 | -0.454** | -0.037 | -0.056 | -0.333* | -0.036 | -0.063 | -0.265 | -0.053 |
|  | (0.15) | (0.18) | (0.15) | (0.15) | (0.17) | (0.15) | (0.14) | (0.17) | (0.14) |
| Accessibility home: 2 stars | 0.140 | -0.487*** | 0.059 | 0.038 | -0.411** | 0.067 | 0.032 | -0.340** | 0.056 |
|  | (0.14) | (0.17) | (0.14) | (0.14) | (0.16) | (0.14) | (0.13) | (0.16) | (0.13) |
| Accessibility home: 3 stars | 0.202 | -0.538*** | 0.018 | 0.113 | -0.480*** | 0.029 | 0.086 | -0.443*** | 0.018 |
|  | (0.14) | (0.17) | (0.14) | (0.14) | (0.16) | (0.14) | (0.13) | (0.16) | (0.13) |
| Constant | -7.183*** | -9.596*** | -6.811*** | -6.747*** | -9.062*** | -6.853*** | -6.443*** | -8.278*** | -6.748*** |
|  | (0.28) | (0.42) | (0.26) | (0.27) | (0.39) | (0.26) | (0.26) | (0.36) | (0.25) |
|  |  |  |  |  |  |  |  |  |  |
| Pseudo R squared | 0.0912 | 0.0912 | 0.0912 | 0.0911 | 0.0911 | 0.0911 | 0.0882 | 0.0882 | 0.0882 |
| Log likelihood | -18859 | -18859 | -18859 | -19794 | -19794 | -19794 | -20569 | -20569 | -20569 |

Regression coefficients for the stroke sample at *T* weeks after the condition took place. *** p<0.01, ** p<0.05, * p<0.1.

|  | T=28 | T=28 | T=28 | T=32 | T=32 | T=32 | T=36 | T=36 | T=36 |
| --- | --- | --- | --- | --- | --- | --- | --- | --- | --- |
|  | Home care | Institutional care | Being deceased | Home care | Institutional care | Being deceased | Home care | Institutional care | Being deceased |
| Healthcare costs quintile 2 | -0.088 | -0.238** | -0.107 | -0.088 | -0.238** | -0.085 | -0.062 | -0.234** | -0.098 |
|  | (0.07) | (0.10) | (0.07) | (0.07) | (0.10) | (0.07) | (0.07) | (0.09) | (0.07) |
| Healthcare costs quintile 3 | -0.001 | -0.435*** | -0.071 | -0.006 | -0.396*** | -0.060 | 0.003 | -0.424*** | -0.065 |
|  | (0.07) | (0.10) | (0.07) | (0.07) | (0.10) | (0.07) | (0.07) | (0.10) | (0.07) |
| Healthcare costs quintile 4 | 0.171** | -0.451*** | 0.047 | 0.197*** | -0.469*** | 0.086 | 0.245*** | -0.421*** | 0.103 |
|  | (0.07) | (0.10) | (0.07) | (0.07) | (0.10) | (0.07) | (0.07) | (0.10) | (0.06) |
| Healthcare costs quintile 5 | 0.516*** | 0.633*** | 0.438*** | 0.525*** | 0.587*** | 0.472*** | 0.532*** | 0.570*** | 0.480*** |
|  | (0.07) | (0.09) | (0.06) | (0.07) | (0.09) | (0.06) | (0.07) | (0.09) | (0.06) |
| Eligibility for long-term care t-14 | 0.364*** | 0.622*** | 0.707*** | 0.330*** | 0.630*** | 0.731*** | 0.365*** | 0.590*** | 0.754*** |
|  | (0.09) | (0.11) | (0.08) | (0.09) | (0.11) | (0.08) | (0.09) | (0.11) | (0.08) |
| Man | -0.203*** | -0.277*** | 0.100** | -0.202*** | -0.282*** | 0.109*** | -0.209*** | -0.246*** | 0.112*** |
|  | (0.04) | (0.06) | (0.04) | (0.04) | (0.06) | (0.04) | (0.04) | (0.06) | (0.04) |
| Partner | -0.532*** | -0.642*** | -0.163*** | -0.527*** | -0.623*** | -0.172*** | -0.518*** | -0.645*** | -0.175*** |
|  | (0.05) | (0.07) | (0.05) | (0.05) | (0.07) | (0.05) | (0.05) | (0.07) | (0.05) |
| Children living in the household | 0.141* | 0.198* | 0.155** | 0.119 | 0.179 | 0.146** | 0.091 | 0.248** | 0.155** |
|  | (0.08) | (0.12) | (0.07) | (0.08) | (0.12) | (0.07) | (0.08) | (0.11) | (0.07) |
| Number of children | 0.024 | -0.021 | -0.032* | 0.033** | -0.011 | -0.029* | 0.039** | -0.013 | -0.032** |
|  | (0.02) | (0.02) | (0.02) | (0.02) | (0.02) | (0.02) | (0.02) | (0.02) | (0.02) |
| Having children | -0.215*** | -0.283*** | -0.188** | -0.241*** | -0.316*** | -0.199*** | -0.241*** | -0.292*** | -0.188*** |
|  | (0.08) | (0.10) | (0.07) | (0.08) | (0.10) | (0.07) | (0.08) | (0.10) | (0.07) |
| First generation immigrant | -0.153* | 0.202** | -0.011 | -0.154* | 0.224** | -0.024 | -0.191** | 0.167* | -0.046 |
|  | (0.08) | (0.10) | (0.08) | (0.08) | (0.10) | (0.07) | (0.08) | (0.10) | (0.07) |
| Second generation immigrant | -0.096 | -0.016 | -0.104 | -0.057 | 0.076 | -0.113 | -0.011 | 0.098 | -0.097 |
|  | (0.09) | (0.13) | (0.09) | (0.09) | (0.13) | (0.09) | (0.09) | (0.12) | (0.09) |
| Age | 0.069*** | 0.090*** | 0.071*** | 0.066*** | 0.086*** | 0.071*** | 0.065*** | 0.085*** | 0.072*** |
|  | (0.00) | (0.00) | (0.00) | (0.00) | (0.00) | (0.00) | (0.00) | (0.00) | (0.00) |
| Urbanity category 2 | -0.007 | -0.033 | -0.026 | 0.004 | -0.070 | -0.013 | -0.014 | -0.057 | -0.010 |
|  | (0.07) | (0.10) | (0.07) | (0.07) | (0.10) | (0.07) | (0.07) | (0.09) | (0.07) |
| Urbanity category 3 | -0.081 | -0.080 | -0.054 | -0.053 | -0.094 | -0.038 | -0.069 | -0.075 | -0.029 |
|  | (0.08) | (0.11) | (0.08) | (0.08) | (0.11) | (0.08) | (0.08) | (0.11) | (0.07) |
| Urbanity category 4 | 0.023 | 0.025 | -0.046 | 0.060 | 0.036 | -0.040 | 0.062 | 0.089 | -0.033 |
|  | (0.09) | (0.12) | (0.08) | (0.09) | (0.12) | (0.08) | (0.09) | (0.12) | (0.08) |
| Urbanity category 5 | 0.177** | 0.083 | -0.020 | 0.208** | 0.084 | 0.020 | 0.213** | 0.083 | 0.028 |
|  | (0.09) | (0.13) | (0.09) | (0.09) | (0.13) | (0.08) | (0.09) | (0.13) | (0.08) |
| Municipality size 2 | -0.375** | -0.403* | 0.139 | -0.310* | -0.308 | 0.136 | -0.300* | -0.192 | 0.135 |
|  | (0.18) | (0.22) | (0.14) | (0.17) | (0.22) | (0.14) | (0.17) | (0.21) | (0.14) |
| Municipality size 3 | 0.193* | -0.115 | 0.180* | 0.192* | -0.030 | 0.176* | 0.166 | -0.005 | 0.205** |
|  | (0.11) | (0.14) | (0.10) | (0.11) | (0.14) | (0.10) | (0.11) | (0.14) | (0.10) |
| Municipality size 4 | 0.244** | -0.161 | 0.064 | 0.178* | -0.133 | 0.048 | 0.128 | -0.163 | 0.049 |
|  | (0.10) | (0.13) | (0.10) | (0.10) | (0.13) | (0.09) | (0.10) | (0.13) | (0.09) |
| Municipality size 5 | 0.177** | -0.224* | 0.162* | 0.154* | -0.145 | 0.132 | 0.128 | -0.125 | 0.135 |
|  | (0.09) | (0.12) | (0.08) | (0.09) | (0.12) | (0.08) | (0.09) | (0.12) | (0.08) |
| Municipality size 6 | 0.238** | -0.184 | 0.107 | 0.193** | -0.125 | 0.078 | 0.153* | -0.120 | 0.078 |
|  | (0.09) | (0.12) | (0.09) | (0.09) | (0.12) | (0.09) | (0.09) | (0.12) | (0.09) |
| Municipality size 7 | 0.099 | -0.197 | 0.103 | 0.032 | -0.181 | 0.065 | -0.008 | -0.174 | 0.064 |
|  | (0.10) | (0.14) | (0.10) | (0.10) | (0.14) | (0.10) | (0.10) | (0.14) | (0.10) |
| Gross income quintile 2 | -0.175*** | -0.224** | -0.083 | -0.183*** | -0.253*** | -0.074 | -0.174*** | -0.244*** | -0.077 |
|  | (0.06) | (0.09) | (0.06) | (0.06) | (0.09) | (0.06) | (0.06) | (0.09) | (0.06) |
| Gross income quintile 3 | -0.456*** | -0.344*** | -0.115* | -0.490*** | -0.309*** | -0.119* | -0.494*** | -0.319*** | -0.130** |
|  | (0.07) | (0.10) | (0.07) | (0.07) | (0.09) | (0.07) | (0.07) | (0.09) | (0.07) |
| Gross income quintile 4 | -0.591*** | -0.382*** | -0.069 | -0.599*** | -0.420*** | -0.069 | -0.638*** | -0.422*** | -0.082 |
|  | (0.08) | (0.11) | (0.07) | (0.08) | (0.10) | (0.07) | (0.08) | (0.10) | (0.07) |
| Gross income quintile 5 | -0.681*** | -0.651*** | -0.098 | -0.651*** | -0.652*** | -0.093 | -0.615*** | -0.698*** | -0.106 |
|  | (0.09) | (0.14) | (0.08) | (0.09) | (0.13) | (0.08) | (0.09) | (0.13) | (0.08) |
| Home ownership | -0.076 | 0.161 | -0.121* | -0.052 | 0.116 | -0.121* | -0.039 | 0.094 | -0.106 |
|  | (0.07) | (0.12) | (0.07) | (0.07) | (0.11) | (0.07) | (0.07) | (0.11) | (0.07) |
| Value of the house | -0.000** | -0.000*** | -0.000** | -0.000*** | -0.000*** | -0.000** | -0.000*** | -0.000*** | -0.000*** |
|  | (0.00) | (0.00) | (0.00) | (0.00) | (0.00) | (0.00) | (0.00) | (0.00) | (0.00) |
| Financial wealth quintile 2 | -0.013 | 0.056 | 0.036 | -0.032 | 0.081 | 0.037 | 0.002 | 0.081 | 0.027 |
|  | (0.07) | (0.10) | (0.07) | (0.07) | (0.09) | (0.07) | (0.07) | (0.09) | (0.06) |
| Financial wealth quintile 3 | 0.011 | 0.059 | -0.063 | -0.060 | 0.095 | -0.074 | -0.005 | 0.053 | -0.089 |
|  | (0.07) | (0.10) | (0.07) | (0.07) | (0.10) | (0.07) | (0.07) | (0.10) | (0.07) |
| Financial wealth quintile 4 | -0.071 | 0.093 | 0.011 | -0.099 | 0.132 | 0.024 | -0.090 | 0.067 | 0.010 |
|  | (0.07) | (0.11) | (0.07) | (0.07) | (0.10) | (0.07) | (0.07) | (0.10) | (0.07) |
| Financial wealth quintile 5 | 0.097 | 0.288** | 0.196** | 0.050 | 0.271** | 0.184** | 0.084 | 0.254** | 0.161** |
|  | (0.08) | (0.12) | (0.08) | (0.08) | (0.12) | (0.08) | (0.08) | (0.12) | (0.08) |
| Accessibility home: 0/3 stars | -0.114 | -0.112 | -0.073 | -0.110 | -0.090 | -0.120 | -0.094 | -0.249 | -0.189 |
|  | (0.14) | (0.17) | (0.14) | (0.14) | (0.17) | (0.14) | (0.14) | (0.16) | (0.13) |
| Accessibility home: 2 stars | 0.019 | -0.223 | 0.017 | -0.012 | -0.208 | -0.038 | 0.008 | -0.344** | -0.118 |
|  | (0.13) | (0.16) | (0.13) | (0.13) | (0.16) | (0.13) | (0.13) | (0.15) | (0.12) |
| Accessibility home: 3 stars | 0.058 | -0.341** | -0.027 | 0.013 | -0.275* | -0.087 | 0.063 | -0.394** | -0.176 |
|  | (0.13) | (0.16) | (0.13) | (0.13) | (0.16) | (0.13) | (0.13) | (0.15) | (0.12) |
| Constant | -6.283*** | -7.790*** | -6.767*** | -5.974*** | -7.579*** | -6.675*** | -5.870*** | -7.337*** | -6.628*** |
|  | (0.26) | (0.35) | (0.25) | (0.25) | (0.34) | (0.24) | (0.25) | (0.34) | (0.24) |
|  |  |  |  |  |  |  |  |  |  |
| Pseudo R squared | 0.0875 | 0.0875 | 0.0875 | 0.0853 | 0.0853 | 0.0853 | 0.0848 | 0.0848 | 0.0848 |
| Log likelihood | -21103 | -21103 | -21103 | -21508 | -21508 | -21508 | -21754 | -21754 | -21754 |

Regression coefficients for the stroke sample at *T* weeks after the condition took place. *** p<0.01, ** p<0.05, * p<0.1.

|  | T=40 | T=40 | T=40 | T=44 | T=44 | T=44 | T=48 | T=48 | T=48 |
| --- | --- | --- | --- | --- | --- | --- | --- | --- | --- |
|  | Home care | Institutional care | Being deceased | Home care | Institutional care | Being deceased | Home care | Institutional care | Being deceased |
| Healthcare costs quintile 2 | -0.052 | -0.226** | -0.084 | -0.071 | -0.216** | -0.103 | -0.092 | -0.214** | -0.103 |
|  | (0.07) | (0.09) | (0.07) | (0.07) | (0.10) | (0.06) | (0.07) | (0.10) | (0.06) |
| Healthcare costs quintile 3 | 0.026 | -0.388*** | -0.064 | 0.018 | -0.347*** | -0.077 | 0.019 | -0.334*** | -0.067 |
|  | (0.07) | (0.10) | (0.07) | (0.07) | (0.10) | (0.06) | (0.07) | (0.10) | (0.06) |
| Healthcare costs quintile 4 | 0.263*** | -0.416*** | 0.116* | 0.242*** | -0.394*** | 0.113* | 0.211*** | -0.322*** | 0.133** |
|  | (0.07) | (0.10) | (0.06) | (0.07) | (0.10) | (0.06) | (0.07) | (0.10) | (0.06) |
| Healthcare costs quintile 5 | 0.549*** | 0.596*** | 0.503*** | 0.541*** | 0.601*** | 0.509*** | 0.544*** | 0.607*** | 0.533*** |
|  | (0.07) | (0.09) | (0.06) | (0.07) | (0.09) | (0.06) | (0.07) | (0.09) | (0.06) |
| Eligibility for long-term care t-14 | 0.323*** | 0.540*** | 0.759*** | 0.242*** | 0.556*** | 0.744*** | 0.214** | 0.572*** | 0.741*** |
|  | (0.09) | (0.11) | (0.08) | (0.09) | (0.11) | (0.08) | (0.09) | (0.11) | (0.08) |
| Man | -0.228*** | -0.220*** | 0.122*** | -0.231*** | -0.229*** | 0.136*** | -0.245*** | -0.198*** | 0.141*** |
|  | (0.04) | (0.06) | (0.04) | (0.04) | (0.06) | (0.04) | (0.04) | (0.06) | (0.04) |
| Partner | -0.503*** | -0.655*** | -0.193*** | -0.506*** | -0.652*** | -0.207*** | -0.487*** | -0.678*** | -0.203*** |
|  | (0.05) | (0.07) | (0.05) | (0.05) | (0.07) | (0.05) | (0.05) | (0.07) | (0.05) |
| Children living in the household | 0.093 | 0.233** | 0.175** | 0.134 | 0.218* | 0.178** | 0.186** | 0.216* | 0.169** |
|  | (0.08) | (0.11) | (0.07) | (0.08) | (0.11) | (0.07) | (0.08) | (0.11) | (0.07) |
| Number of children | 0.033** | -0.017 | -0.032** | 0.033** | -0.013 | -0.031* | 0.039** | -0.009 | -0.032** |
|  | (0.02) | (0.02) | (0.02) | (0.02) | (0.02) | (0.02) | (0.02) | (0.02) | (0.02) |
| Having children | -0.230*** | -0.271*** | -0.187*** | -0.212*** | -0.275*** | -0.172** | -0.247*** | -0.350*** | -0.173** |
|  | (0.08) | (0.10) | (0.07) | (0.08) | (0.10) | (0.07) | (0.08) | (0.10) | (0.07) |
| First generation immigrant | -0.206** | 0.177* | -0.044 | -0.175** | 0.178* | -0.042 | -0.208** | 0.149 | -0.045 |
|  | (0.08) | (0.10) | (0.07) | (0.08) | (0.10) | (0.07) | (0.08) | (0.10) | (0.07) |
| Second generation immigrant | 0.027 | 0.109 | -0.068 | 0.005 | 0.093 | -0.084 | -0.015 | 0.132 | -0.076 |
|  | (0.09) | (0.12) | (0.09) | (0.09) | (0.12) | (0.09) | (0.09) | (0.12) | (0.09) |
| Age | 0.065*** | 0.085*** | 0.072*** | 0.065*** | 0.085*** | 0.073*** | 0.064*** | 0.086*** | 0.074*** |
|  | (0.00) | (0.00) | (0.00) | (0.00) | (0.00) | (0.00) | (0.00) | (0.00) | (0.00) |
| Urbanity category 2 | 0.017 | -0.043 | 0.001 | 0.014 | -0.010 | -0.022 | 0.009 | -0.031 | -0.040 |
|  | (0.07) | (0.09) | (0.06) | (0.07) | (0.09) | (0.06) | (0.07) | (0.09) | (0.06) |
| Urbanity category 3 | -0.032 | -0.057 | -0.026 | 0.004 | -0.034 | -0.030 | -0.014 | -0.025 | -0.038 |
|  | (0.08) | (0.11) | (0.07) | (0.08) | (0.11) | (0.07) | (0.08) | (0.11) | (0.07) |
| Urbanity category 4 | 0.089 | 0.068 | -0.024 | 0.120 | 0.051 | -0.015 | 0.123 | 0.014 | -0.010 |
|  | (0.09) | (0.12) | (0.08) | (0.09) | (0.12) | (0.08) | (0.09) | (0.12) | (0.08) |
| Urbanity category 5 | 0.261*** | 0.049 | 0.052 | 0.237*** | 0.075 | 0.040 | 0.213** | 0.058 | 0.031 |
|  | (0.09) | (0.13) | (0.08) | (0.09) | (0.13) | (0.08) | (0.09) | (0.13) | (0.08) |
| Municipality size 2 | -0.333** | -0.383* | 0.160 | -0.347** | -0.318 | 0.143 | -0.265 | -0.313 | 0.124 |
|  | (0.17) | (0.22) | (0.14) | (0.17) | (0.21) | (0.13) | (0.16) | (0.21) | (0.13) |
| Municipality size 3 | 0.179* | -0.039 | 0.212** | 0.167 | -0.017 | 0.200** | 0.201* | -0.019 | 0.198** |
|  | (0.11) | (0.14) | (0.10) | (0.11) | (0.14) | (0.10) | (0.10) | (0.14) | (0.10) |
| Municipality size 4 | 0.153 | -0.175 | 0.070 | 0.152 | -0.126 | 0.061 | 0.110 | -0.113 | 0.044 |
|  | (0.10) | (0.13) | (0.09) | (0.10) | (0.13) | (0.09) | (0.10) | (0.13) | (0.09) |
| Municipality size 5 | 0.113 | -0.163 | 0.168** | 0.105 | -0.128 | 0.152* | 0.068 | -0.149 | 0.141* |
|  | (0.09) | (0.12) | (0.08) | (0.09) | (0.12) | (0.08) | (0.09) | (0.12) | (0.08) |
| Municipality size 6 | 0.139 | -0.143 | 0.098 | 0.161* | -0.144 | 0.091 | 0.114 | -0.114 | 0.087 |
|  | (0.09) | (0.12) | (0.09) | (0.09) | (0.12) | (0.08) | (0.09) | (0.12) | (0.08) |
| Municipality size 7 | 0.009 | -0.211 | 0.074 | -0.009 | -0.180 | 0.067 | -0.006 | -0.174 | 0.071 |
|  | (0.10) | (0.14) | (0.10) | (0.10) | (0.14) | (0.09) | (0.10) | (0.14) | (0.09) |
| Gross income quintile 2 | -0.192*** | -0.230*** | -0.080 | -0.198*** | -0.211** | -0.069 | -0.225*** | -0.172** | -0.067 |
|  | (0.06) | (0.09) | (0.06) | (0.06) | (0.09) | (0.06) | (0.06) | (0.08) | (0.06) |
| Gross income quintile 3 | -0.496*** | -0.382*** | -0.135** | -0.464*** | -0.376*** | -0.133** | -0.458*** | -0.363*** | -0.146** |
|  | (0.07) | (0.09) | (0.07) | (0.07) | (0.09) | (0.06) | (0.07) | (0.09) | (0.06) |
| Gross income quintile 4 | -0.645*** | -0.454*** | -0.103 | -0.688*** | -0.427*** | -0.098 | -0.703*** | -0.428*** | -0.098 |
|  | (0.08) | (0.10) | (0.07) | (0.08) | (0.10) | (0.07) | (0.08) | (0.10) | (0.07) |
| Gross income quintile 5 | -0.637*** | -0.680*** | -0.142* | -0.655*** | -0.612*** | -0.149* | -0.688*** | -0.599*** | -0.154* |
|  | (0.09) | (0.13) | (0.08) | (0.09) | (0.13) | (0.08) | (0.09) | (0.13) | (0.08) |
| Home ownership | -0.030 | 0.175 | -0.121* | -0.016 | 0.127 | -0.116* | -0.025 | 0.128 | -0.114* |
|  | (0.07) | (0.11) | (0.07) | (0.07) | (0.11) | (0.07) | (0.07) | (0.11) | (0.07) |
| Value of the house | -0.000** | -0.000*** | -0.000** | -0.000*** | -0.000*** | -0.000** | -0.000*** | -0.000*** | -0.000*** |
|  | (0.00) | (0.00) | (0.00) | (0.00) | (0.00) | (0.00) | (0.00) | (0.00) | (0.00) |
| Financial wealth quintile 2 | -0.016 | 0.092 | 0.038 | -0.021 | 0.062 | 0.038 | -0.016 | 0.038 | 0.031 |
|  | (0.07) | (0.09) | (0.06) | (0.07) | (0.09) | (0.06) | (0.07) | (0.09) | (0.06) |
| Financial wealth quintile 3 | -0.001 | 0.046 | -0.064 | -0.031 | 0.036 | -0.071 | -0.033 | 0.007 | -0.062 |
|  | (0.07) | (0.10) | (0.07) | (0.07) | (0.10) | (0.07) | (0.07) | (0.10) | (0.07) |
| Financial wealth quintile 4 | -0.116 | 0.053 | 0.038 | -0.158** | 0.012 | 0.046 | -0.155** | 0.020 | 0.046 |
|  | (0.07) | (0.10) | (0.07) | (0.07) | (0.10) | (0.07) | (0.07) | (0.10) | (0.07) |
| Financial wealth quintile 5 | 0.028 | 0.231** | 0.182** | 0.002 | 0.224* | 0.173** | 0.011 | 0.220* | 0.173** |
|  | (0.08) | (0.11) | (0.07) | (0.08) | (0.11) | (0.07) | (0.08) | (0.11) | (0.07) |
| Accessibility home: 0/3 stars | -0.148 | -0.257 | -0.199 | -0.146 | -0.200 | -0.187 | -0.173 | -0.043 | -0.223* |
|  | (0.14) | (0.16) | (0.13) | (0.14) | (0.17) | (0.13) | (0.14) | (0.17) | (0.13) |
| Accessibility home: 2 stars | -0.004 | -0.299* | -0.132 | -0.004 | -0.242 | -0.113 | -0.040 | -0.154 | -0.133 |
|  | (0.13) | (0.16) | (0.12) | (0.13) | (0.16) | (0.12) | (0.13) | (0.16) | (0.12) |
| Accessibility home: 3 stars | 0.039 | -0.333** | -0.200 | 0.081 | -0.298* | -0.166 | 0.060 | -0.180 | -0.194 |
|  | (0.13) | (0.16) | (0.12) | (0.13) | (0.16) | (0.12) | (0.13) | (0.16) | (0.12) |
| Constant | -5.859*** | -7.382*** | -6.571*** | -5.843*** | -7.463*** | -6.628*** | -5.655*** | -7.588*** | -6.632*** |
|  | (0.25) | (0.34) | (0.24) | (0.25) | (0.34) | (0.23) | (0.25) | (0.34) | (0.23) |
|  |  |  |  |  |  |  |  |  |  |
| Pseudo R squared | 0.0841 | 0.0841 | 0.0841 | 0.0846 | 0.0846 | 0.0846 | 0.0850 | 0.0850 | 0.0850 |
| Log likelihood | -21960 | -21960 | -21960 | -22119 | -22119 | -22119 | -22261 | -22261 | -22261 |

Regression coefficients for the stroke sample at *T* weeks after the condition took place. *** p<0.01, ** p<0.05, * p<0.1.

|  | T=52 | T=52 | T=52 |
| --- | --- | --- | --- |
|  | Home care | Institutional care | Being deceased |
| Healthcare costs quintile 2 | -0.104 | -0.218** | -0.093 |
|  | (0.07) | (0.10) | (0.06) |
| Healthcare costs quintile 3 | -0.002 | -0.311*** | -0.065 |
|  | (0.07) | (0.10) | (0.06) |
| Healthcare costs quintile 4 | 0.222*** | -0.339*** | 0.147** |
|  | (0.07) | (0.10) | (0.06) |
| Healthcare costs quintile 5 | 0.537*** | 0.594*** | 0.561*** |
|  | (0.07) | (0.09) | (0.06) |
| Eligibility for long-term care t-14 | 0.254*** | 0.626*** | 0.751*** |
|  | (0.09) | (0.11) | (0.08) |
| Man | -0.227*** | -0.184*** | 0.145*** |
|  | (0.04) | (0.06) | (0.04) |
| Partner | -0.460*** | -0.666*** | -0.198*** |
|  | (0.05) | (0.07) | (0.05) |
| Children living in the household | 0.137* | 0.183 | 0.177** |
|  | (0.08) | (0.12) | (0.07) |
| Number of children | 0.040** | -0.003 | -0.035** |
|  | (0.02) | (0.02) | (0.02) |
| Having children | -0.235*** | -0.342*** | -0.175** |
|  | (0.08) | (0.10) | (0.07) |
| First generation immigrant | -0.220*** | 0.137 | -0.070 |
|  | (0.08) | (0.10) | (0.07) |
| Second generation immigrant | -0.005 | 0.121 | -0.079 |
|  | (0.09) | (0.12) | (0.08) |
| Age | 0.064*** | 0.086*** | 0.074*** |
|  | (0.00) | (0.00) | (0.00) |
| Urbanity category 2 | 0.017 | -0.070 | -0.040 |
|  | (0.07) | (0.09) | (0.06) |
| Urbanity category 3 | -0.022 | -0.053 | -0.047 |
|  | (0.08) | (0.11) | (0.07) |
| Urbanity category 4 | 0.092 | 0.007 | -0.022 |
|  | (0.09) | (0.12) | (0.08) |
| Urbanity category 5 | 0.210** | 0.021 | 0.020 |
|  | (0.09) | (0.13) | (0.08) |
| Municipality size 2 | -0.301* | -0.313 | 0.112 |
|  | (0.16) | (0.22) | (0.13) |
| Municipality size 3 | 0.146 | 0.025 | 0.162* |
|  | (0.10) | (0.14) | (0.10) |
| Municipality size 4 | 0.108 | -0.077 | 0.032 |
|  | (0.10) | (0.13) | (0.09) |
| Municipality size 5 | 0.059 | -0.092 | 0.137* |
|  | (0.09) | (0.12) | (0.08) |
| Municipality size 6 | 0.097 | -0.060 | 0.078 |
|  | (0.09) | (0.12) | (0.08) |
| Municipality size 7 | -0.012 | -0.122 | 0.058 |
|  | (0.10) | (0.14) | (0.09) |
| Gross income quintile 2 | -0.226*** | -0.159* | -0.081 |
|  | (0.06) | (0.08) | (0.06) |
| Gross income quintile 3 | -0.489*** | -0.359*** | -0.159** |
|  | (0.07) | (0.09) | (0.06) |
| Gross income quintile 4 | -0.696*** | -0.445*** | -0.111 |
|  | (0.08) | (0.10) | (0.07) |
| Gross income quintile 5 | -0.741*** | -0.590*** | -0.164** |
|  | (0.09) | (0.13) | (0.08) |
| Home ownership | -0.045 | 0.138 | -0.108* |
|  | (0.07) | (0.11) | (0.06) |
| Value of the house | -0.000** | -0.000*** | -0.000*** |
|  | (0.00) | (0.00) | (0.00) |
| Financial wealth quintile 2 | -0.076 | 0.009 | 0.032 |
|  | (0.06) | (0.09) | (0.06) |
| Financial wealth quintile 3 | -0.096 | 0.026 | -0.060 |
|  | (0.07) | (0.10) | (0.06) |
| Financial wealth quintile 4 | -0.238*** | -0.008 | 0.034 |
|  | (0.07) | (0.10) | (0.07) |
| Financial wealth quintile 5 | -0.030 | 0.193* | 0.160** |
|  | (0.08) | (0.11) | (0.07) |
| Accessibility home: 0/3 stars | -0.163 | -0.070 | -0.186 |
|  | (0.14) | (0.17) | (0.13) |
| Accessibility home: 2 stars | -0.053 | -0.211 | -0.104 |
|  | (0.13) | (0.16) | (0.12) |
| Accessibility home: 3 stars | 0.049 | -0.240 | -0.165 |
|  | (0.13) | (0.16) | (0.12) |
| Constant | -5.574*** | -7.516*** | -6.649*** |
|  | (0.25) | (0.34) | (0.23) |
|  |  |  |  |
| Pseudo R squared | 0.0852 | 0.0852 | 0.0852 |
| Log likelihood | -22452 | -22452 | -22452 |

Regression coefficients for the stroke sample at *T* weeks after the condition took place. *** p<0.01, ** p<0.05, * p<0.1.
